# Differential Effects of Regulatory T Cells in the Meninges and Spinal Cord of Male and Female Mice with Neuropathic Pain

**DOI:** 10.3390/cells12182317

**Published:** 2023-09-20

**Authors:** Nathan T. Fiore, Brooke A. Keating, Yuting Chen, Sarah I. Williams, Gila Moalem-Taylor

**Affiliations:** Translational Neuroscience Facility, Department of Physiology, School of Biomedical Sciences, University of New South Wales (UNSW), Sydney, NSW 2052, Australia; ntfiore@mdanderson.org (N.T.F.); brooke.keating@health.nsw.gov.au (B.A.K.); yuting.chen4@student.unsw.edu.au (Y.C.); sarahilwil@gmail.com (S.I.W.)

**Keywords:** peripheral nerve injury, neuroimmune, regulatory T cells, neuropathic pain, sex differences

## Abstract

Immune cells play a critical role in promoting neuroinflammation and the development of neuropathic pain. However, some subsets of immune cells are essential for pain resolution. Among them are regulatory T cells (Tregs), a specialised subpopulation of T cells that limit excessive immune responses and preserve immune homeostasis. In this study, we utilised intrathecal adoptive transfer of activated Tregs in male and female mice after peripheral nerve injury to investigate Treg migration and whether Treg-mediated suppression of pain behaviours is associated with changes in peripheral immune cell populations in lymphoid and meningeal tissues and spinal microglial and astrocyte reactivity and phenotypes. Treatment with Tregs suppressed mechanical pain hypersensitivity and improved changes in exploratory behaviours after chronic constriction injury (CCI) of the sciatic nerve in both male and female mice. The injected Treg cells were detected in the choroid plexus and the pia mater and in peripheral lymphoid organs in both male and female recipient mice. Nonetheless, Treg treatment resulted in differential changes in meningeal and lymph node immune cell profiles in male and female mice. Moreover, in male mice, adoptive transfer of Tregs ameliorated the CCI-induced increase in microglia reactivity and inflammatory phenotypic shift, increasing M2-like phenotypic markers and attenuating astrocyte reactivity and neurotoxic astrocytes. Contrastingly, in CCI female mice, Treg injection increased astrocyte reactivity and neuroprotective astrocytes. These findings show that the adoptive transfer of Tregs modulates meningeal and peripheral immunity, as well as spinal glial populations, and alleviates neuropathic pain, potentially through different mechanisms in males and females.

## 1. Introduction

Neuropathic pain, “pain caused by a lesion or disease of the somatosensory nervous system” [1,2], is widely accepted as one of the most challenging forms of pain to treat effectively, severely impacting patient quality of life [3,4]. Clinical presentation of neuropathic pain may be highly variable and include negative symptoms, such as sensory loss and numbness, and positive symptoms, such as paraesthesia, spontaneous pain, and allodynia. A growing body of evidence indicates a dysregulated immune response resulting in a proinflammatory cellular milieu in neural pain pathways is heavily involved in the pathophysiology of neuropathic pain [5,6,7,8]. Although many components of the innate and adaptive immune systems have been shown to promote neuroinflammation and contribute to pain hypersensitivity, recent studies have highlighted several immune cell subpopulations important for pain resolution [9].

Regulatory T cells (Tregs) are a specialised class of cells that belong to the adaptive immune system and are essential for preventing autoimmune and inflammatory pathologies. Tregs can modulate innate and adaptive immune responses, limiting inflammation and preserving immune homeostasis through mechanisms including metabolic disruption, modulation of dendritic cell (DC) maturation, direct cytotoxicity, and the production of anti-inflammatory cytokines [10]. Moreover, Tregs have been implicated in tissue maintenance, repair, and regeneration, such as promoting central nervous system (CNS) remyelination [11,12]. Tregs have been shown to contribute to pain resolution in rodent models of neuropathic pain [9,13]. Increasing Tregs by various methods reduces pain hypersensitivity in models of peripheral nerve injury [14,15,16], experimental autoimmune neuritis [14], paclitaxel-induced peripheral neuropathy [17], morphine-resistant mechanical allodynia [18], and experimental autoimmune encephalomyelitis (EAE) [12] due to the secretion of inhibitory cytokines such as interleukin (IL)-10 and IL-35, downregulation of type 1 helper T (Th1) cells [16], and increasing M2-like macrophages [19]. Treg depletion increases mechanical pain hypersensitivity [20] and delays recovery after peripheral nerve injury in both males and females [15]. Additionally, the downregulation of Tregs is associated with chronic muscle pain pathology in a mouse model of fibromyalgia [21].

Sex differences in pain studies are now well established. Evidence indicates that women are more likely to suffer from chronic pain [22] and report higher pain levels than men across diverse diseases, ages, and geographical regions [23]. Although the mechanisms underlying these sex differences are not fully understood, recent studies have revealed a sex-specific involvement of different genes, proteins, hormones, and cellular responses with distinct effects on immune function, influencing pain signalling [24]. For example, activating Toll-like receptor 4 (TLR4) in the spinal cord caused mechanical allodynia only in male mice, and inflammatory and neuropathic pain behaviours depended on TLR4 in males but not females [25]. Furthermore, although males and females have equivalent spinal microglia activation after peripheral nerve injury, male mice preferentially rely on microglia and female mice on T cells to mediate mechanical pain hypersensitivity [26]. A recent study suggested a potential explanation for the sexual dimorphism in microglial contribution to pain signalling, demonstrating that Tregs restrict microglial activation and pain behaviour following intrathecal injection of colony-stimulating factor-1 (CSF-1) in female, but not male, mice through Treg expansion within the spinal cord meninges [27].

The CNS anatomical borders, including the meninges and the blood-cerebrospinal fluid barrier (BCSFB), have recently been recognised as highly active immune interfaces between the CNS and the periphery, housing various resident immune cells that control CNS function [28]. The meninges are membranes that line the skull and vertebral canal, enclosing the brain and spinal cord and containing the outermost dura mater, the middle arachnoid mater, and the innermost pia mater that adheres to the CNS parenchyma [29,30]. The recently characterised meningeal lymphatic network plays a critical role in CNS drainage. The dural lymphatic vessels drain interstitial fluid, cerebrospinal fluid (CSF), macromolecules, and immune cells from the brain to the cervical lymph nodes [31]. The vertebral lymphatic vasculature, located in the intervertebral tissue, drains the epidural lymph of the vertebral column to peripheral lymph nodes [32], thus enabling communication between the CNS and peripheral immunity. Similarly, the choroid plexus, a secretory structure within the brain’s ventricles that produces CSF and forms the BCSFB, has also been confirmed as an essential neuroimmunological niche allowing immune cell infiltration in both health and disease [28,33].

The confirmation of immune cell activity of the meninges and choroid plexus and the ability of meningeal Tregs to regulate glial reactivity [27] and affect neuropathic pain experiences [12] presents an attractive therapeutic avenue. However, no study has tracked the migration of donor Tregs following adoptive transfer by intrathecal delivery into the spinal canal, which reaches the CSF. Additionally, how the adoptive transfer of Tregs affects male and female nerve-injured mice remains unknown. Here, we demonstrate that activated Tregs delivered intrathecally are present in the meninges 48 h and up to 6 days following delivery and migrate into the peripheral lymphatic system within this time frame. Treatment with Tregs can ameliorate neuropathic pain behaviours following chronic constriction injury (CCI) of the sciatic nerve in both sexes. However, we show different changes in immune profiles in male and female mice’s meninges, choroid plexus, and peripheral lymph nodes after CCI and intrathecal delivery of activated Tregs. Similarly, activated Tregs regulate spinal glial reactivity in a sex-dependent manner. These results suggest immune responses in the meninges, choroid plexus, peripheral lymph nodes, and spinal cord can be modulated with intrathecal Treg delivery, although to a different extent in male and female mice.

## 2. Materials and Methods

### 2.1. Animals

C57BL/6J or DEREG (depletion of regulatory T cell) mice [34] (Australian Biological Resources, Moss Vale, NSW, Australia) aged 8–12 weeks were used for all experiments (sex stated for each experiment in subsequent sections). Mice were housed in individually ventilated cages and maintained on a 12-h light/dark cycle at constant room temperature and humidity. Food and water were available *ad libitum.* All experiments were approved by the Animal Care and Ethics Committee (ACEC) of UNSW.

### 2.2. EAE Induction

EAE was induced in 9-week-old female C57BL/6J and DEREG mice through subcutaneous immunisation with myelin oligodendrocyte glycoprotein (MOG)_35–55_ emulsified in Complete Freund’s Adjuvant (CFA) as pre-filled syringes containing 1 mg/mL MOG_35–55_ and 2–5 mg killed *Mycobacterium tuberculosis* H37Ra/mL in CFA (Hooke Laboratories, Lawrence, MA, USA). Control mice were immunised subcutaneously with CFA alone. Subcutaneous injections (2 × 100 µL) were performed under 3–5% isoflurane anaesthesia in oxygen and given on both sides of the spinal column on the lower back, approximately 1 cm above the base of the tail (200 µg delivered/animal). An intraperitoneal (i.p.) injection of 200 ng Pertussis toxin (Hooke Laboratories) in 100 µL Dulbecco’s phosphate-buffered saline (D-PBS; Life Technologies Pty Ltd., Tullamarine, VIC, Australia) was given to all mice 2–4 h following immunisation, and again 24 h later. Post-induction, mice were monitored daily for body weight and EAE clinical scores, determined by a grading scale supplied by Hooke Laboratories. Briefly, scores were assigned as: Grade 1 = limp tail; Grade 2 = limp tail and weakness of hind legs; Grade 3 = limp tail and complete paralysis of hind legs or limp tail with paralysis of one front and one hind leg; Grade 4 = limp tail, complete hind leg and partial front leg paralysis; and Grade 5 = complete hind and complete front leg paralysis.

### 2.3. Chronic Constriction Injury

CCI of the left sciatic nerve was performed on both male and female 8-week-old C57BL/6J mice following the procedure outlined previously [35]. Briefly, mice were anaesthetised under 3–5% isoflurane anaesthesia in oxygen, and the left hind limb was shaved at the thigh and sterilised with 70% ethanol and iodine solution. An incision was made through the skin and connective tissue between the gluteus superficialis and the biceps femoris muscles to expose the sciatic nerve. In nerve-injured mice, two chromic gut ligatures (6/0, Ethicon, Raritan, NJ, USA) were placed around the nerve 1 mm apart, tightly enough to occlude but not prevent epineural blood flow. Silk sutures (5/0, Mersilk, Ethicon) were used to close the muscle layer, and wound reflex clips (9 mm, Autoclips, BD Diagnostic, Macquarie Park, NSW, Australia) were used to close the skin. In sham (control) mice, the sciatic nerve was exposed and visualised, but no ligatures were placed, and muscle and skin layers were closed, as described above.

### 2.4. Regulatory T Cell Isolation and Activation

Tregs were isolated from female DEREG mice 30 days following EAE induction (i.e., mice eight weeks old at the time of induction) or male and female mice seven days following CCI. Inguinal and cervical lymph nodes (ILN and CLN, respectively) from EAE or sciatic and popliteal lymph nodes from CCI, along with spleen were removed from animals under 5% isoflurane anaesthesia in oxygen and kept in Hanks’ Balanced Salt Solution (HBSS; Gibco, Scoresby, VIC, Australia), containing 0.5% fetal bovine serum (FBS; Invitrogen, Mulgrave, VIC, Australia), 20 mM 4-(2-hydroxyethyl)-1-piperazineethanesulfonic acid (HEPES; Gibco), and 40 mM penicillin-streptomycin (PS; Invitrogen). Mice were then humanely euthanised with an i.p. injection of Lethabarb (Virbac, Milperra, NSW, Australia). Tissues were mechanically ground through a 40 μm cell strainer (Thermo Fisher Scientific, Ultimo, NSW, Australia) to obtain a single cell suspension and centrifuged at 300× *g* for 5 min at 25 °C, and the supernatant was discarded. Cells were resuspended in 5 mL red blood cell lysis buffer (eBioscience through Thermo Fisher Scientific) and agitated for 5 min at room temperature, after which 8 mL HBSS solution was added. Cells were centrifuged once more at 300× *g* for 5 min at 25 °C, the supernatant was discarded, and cells were washed twice in RPMI (Roswell Park Memorial Institute) media (Invitrogen) plus 5% FBS (Invitrogen). Cells were then resuspended in RPMI plus 5% FBS and incubated at 37 °C/5% CO_2_ for 1 h to remove monocytes. Non-adherent cells were collected, samples were centrifuged at 300× *g* for 5 min at 25 °C, and cells were resuspended in an HBSS solution. Tregs were isolated based on GFP expression using a BD FACSAria III cell sorter (BD Biosciences Australia, North Ryde, NSW, Australia).

Following isolation, Tregs were centrifuged (500× *g* for 3 min at 25 °C), resuspended in 1 mL HBSS solution, and counted using a Countess Automated Cell Counter (Invitrogen). Cells were centrifuged once more (600× *g* for 3 min at 25 °C) and resuspended in complete filtered media containing RPMI (Invitrogen), 5% FBS (Invitrogen), 10 mM HEPES (Gibco), 1% non-essential amino acids solution (Gibco), 1% sodium pyruvate (Gibco), 0.1% β-mercaptoethanol (Gibco), and 1% PS (Invitrogen). Cells were then placed in a 96-well plate (100,000 cells/200 μL), and anti-CD3/CD28 dynabeads (Life Technologies) were added to each well to stimulate Tregs in a 1:1 cell-to-bead ratio. IL-2 (R&D Systems, Minneapolis, MN, USA) was added to each well for a final concentration of 10 ng/200 μL. Cells were then incubated at 37 °C/5% CO_2_ for seven days for activation and proliferation.

### 2.5. Adoptive Transfer of Activated Tregs

Following seven days of activation, Tregs were removed from each well and pooled per experimental group. Anti-CD3/CD28 dynabeads (Life Technologies) were removed through magnetic separation, and cells were counted (Countess, Invitrogen) in 1 mL complete filtered media (see above). Cells were centrifuged (600× *g* for 5 min at 25 °C) and resuspended in filtered D-PBS (Life Technologies) to give a concentration of 500,000 cells per recipient animal in 5 µL. Recipient animals were anaesthetised under 4–5% isoflurane in oxygen, and the hair was removed from the lower back, above the iliac crest. Activated Tregs were delivered via intrathecal injection between the L5/L6 vertebrae (i.e., lumbar puncture), with each recipient male or female mouse receiving an injection of 500,000 cells in 5 µL D-PBS (derived from donor mice of the corresponding sex). Injections were delivered over 30 s. Control animals received 5 µL sterile D-PBS intrathecal, delivered under the same conditions. Animals were then placed on a heated mat (40 °C) and monitored during recovery from anaesthesia before being returned to their home cages.

### 2.6. Behavioural Tests

#### 2.6.1. Von Frey Testing

Mechanical sensitivity was assessed before nerve injury and on days three, six and nine following nerve injury using calibrated von Frey filaments. Mice were habituated to the behavioural testing setup for a minimum of 30 min before collecting data in a quiet and well-controlled environment. The mechanical withdrawal threshold was assessed using the up-down technique. Briefly, mice were introduced into the testing enclosure with a raised mesh floor, and the mid-plantar area was stimulated using a set of 8 calibrated von Frey filaments (0.02, 0.04, 0.07, 0.16, 0.4, 0.6, 1.0 and 1.4 g) until the filament was slightly bent. A positive response was recorded when a withdrawal reflex was observed. The first filament was always 0.4 g; following a positive response, the 0.16 g filament was applied, and following a negative response, the 0.6 g filament was used. Each hindpaw underwent four trials following the initial positive response, with a minimum of 3 min between successive trials on the same hindpaw. The 50% paw withdrawal threshold was then calculated.

#### 2.6.2. Open Field Hole-Board Test

Nine days after the nerve injury, exploratory behaviour testing was conducted using a photobeam activity system (PAS; San Diego Instruments, San Diego, CA, USA) in a controlled environment maintained at a consistent temperature. Mice were positioned at the centre of a PAS chamber measuring 40 cm in width, 40 cm in diameter, and 38 cm in height. The chamber was surrounded by a customised open-top box made of white Perspex, which blocked the view of the room except for the ceiling. Several parameters, including activity, nose-poke, and rearing events, were recorded for 5 min by detecting breaks in the photobeam. The nose-poking behaviour was measured using manufacturer-supplied flooring containing 16 evenly spaced nose-poke holes (hole-board) equipped with laser activation to register each instance of the mice investigating them. The data collected from the photobeam break recordings were analyzed using the manufacturer’s software to determine metrics such as distance travelled, average speed, time spent in the central area of the chamber, and nose-poking and rearing actions.

### 2.7. Tissue Dissection

Mice were anaesthetised under 3–5% isoflurane in oxygen, and ILNs, CLNs, sciatic and popliteal LNs, and spleens were removed and placed in 1 mL of phosphate-buffered saline (PBS) on ice. Mice were then transcardially perfused with ice-cold heparinised 0.9% saline and decapitated. Following decapitation, the skin of the head was removed, as well as any excess muscle and connective tissue, the skull was exposed, and the mandible and the cribriform plate were removed. Then, the brain was removed through the ventral aspect of the skull while leaving the dorsal skull intact. The skull cap and brain of each mouse were then placed into 10 mL ice-cold PBS. Under a dissecting microscope, the dura/arachnoid mater was removed from the inside of the skull cap by scoring the circumference of the skull cap with fine forceps. An incision was made between the cerebellum and the cerebrum until the cerebellum was severed. The cerebellum and brainstem were then gently pulled apart, exposing the fourth ventricle and allowing the choroid plexus to be removed. Two 45° incisions were made across each cerebral hemisphere, allowing the cerebrum to be lifted away from the lateral ventricles. The choroid plexus was removed from the lateral ventricles and pooled with the tissue taken from the fourth ventricle. Once choroid plexus tissue had been collected, the pia mater was slowly removed from the remaining brain parenchyma and the ventral aspect of the cerebellum. In all experiments, dura and arachnoid mater were pooled and analysed together as this technique does not allow for differentiation between the two layers.

All tissue collected was placed into 1 mL PBS on ice. For the CCI experiments, each mouse’s lumbar spinal cord (L3–L5 segment) was removed and postfixed in 4% paraformaldehyde in PBS overnight at 4 °C. The tissues were then stored in 30% sucrose with 0.05% sodium azide solution at 4 °C. Tissue sectioning was performed using a cryostat (Leica Biosystems, Nussloch, Germany). Spinal cord sections were cut in optimal cutting temperature media at a thickness of 10µm and, mounted on SuperFrost Gold slides (ThermoFisher, Waltham, MA, USA) and stored at −30 °C.

### 2.8. Flow Cytometry

Following dissection, lymphoid tissue was mechanically ground through a 40 μm cell strainer (Thermo Fisher Scientific). Strainers were rinsed with 10 mL PBS to obtain a single-cell suspension. Samples were centrifuged at 600× *g* for 5 min at 4 °C, and the supernatant was discarded. ILNs and CLNs were resuspended in 1.5 mL and 3 mL PBS, respectively. Spleens were resuspended in 5 mL red blood cell lysis buffer (eBioscience, San Diego, CA, USA) and agitated at room temperature for 5 min before 8 mL PBS was added. Spleens were centrifuged (600× *g* for 5 min at 4 °C). Samples were resuspended in 8 mL PBS, and 100 μL of each sample was then taken and placed into 1.5 mL Eppendorf tubes for staining.

Following dissection, the PBS in each meningeal sample tube was removed and replaced with 0.5 mL Accutase (Merck, Darmstadt, Germany). Samples were incubated in the enzyme for 30 min at 37 °C/5% CO_2_. Tissue was then mechanically ground through a 70 μm cell strainer (Thermo Fisher Scientific), and strainers were rinsed with 10 mL PBS to obtain a single-cell suspension. Samples were centrifuged (1000× *g* for 5 min at 4 °C), and the supernatant was discarded, leaving ~300 μL at the bottom of each tube. This solution was briefly vortexed and transferred to a 1.5 mL Eppendorf tube for staining.

All samples were incubated with 1 μL/sample Zombie cell viability dye (BioLegend, San Diego, CA, USA) for 30 min at room temperature in the dark. 1 mL autoMACS running buffer (Miltenyi Biotec, Bergisch Gladbach, Germany) was added, samples were centrifuged (600× *g* for 5 min at 4 °C), and the supernatant was discarded. Samples were stained for cell surface markers for 30 min at 4 °C using the following antibodies in 100 μL autoMACS running buffer: anti-CD45-BV510 (BioLegend), anti-CD3-PerCP/Cy5.5 (BioLegend), anti-CD4-BV711 (BioLegend), anti-CD11b-BV650 (BioLegend), anti-CD8a-BV421 (BioLegend), anti-CD25-APC (BioLegend), anti-CD19-PEDazzle (BioLegend), anti-CD11c-PE (BioLegend), anti-CD62L-PECy5 (BioLegend), and anti-CD44-BB515 (BD Biosciences). Samples were washed 3 times with 1 mL autoMACS running buffer and centrifuged at 600× *g* for 5 min at 4 °C. To assess GFP+ Treg trafficking, samples were stained for CD45, CD3, CD4 and CD25 using the above-listed antibodies, then resuspended in 200 μL autoMACS running buffer for analysis. All other samples were incubated overnight at 4 °C in 0.5 mL/sample fixation/permeabilisation buffer (Thermo Fisher Scientific) to allow for intracellular staining.

Following overnight fixation, samples were washed twice in 1 mL 1× permeabilisation buffer (Thermo Fisher Scientific) (centrifuged at 600× *g* for 5 min at 4 °C). Samples were then incubated with 1 μL/sample anti-FoxP3-PECy7 (eBioscience) in 100 μL permeabilisation buffer for 30 min at room temperature in the dark. Samples were washed 3 times with 1 mL permeabilisation buffer and centrifuged at 600× *g* for 5 min at 4 °C. Samples were resuspended in 200 μL autoMACS running buffer. Flow cytometric analysis was performed using an LSRFortessa X20 flow cytometer (BD Biosciences), running FACSDiva 7 software (BD Biosciences). Data were analysed using FlowJo software 10 (FlowJo, Ashland, OR, USA). Appropriate fluorescence-minus-one (FMO) controls were included in each experiment. The gating strategy used for flow cytometric analysis is described in Figure S1.

### 2.9. Opal Multiplex Immunohistochemistry

Opal Multiplex immunohistochemistry was conducted on lumbar spinal cord sections using Leica Bond RXm (Leica Biosystems, Germany), with the Opal 6-colour automation immunohistochemistry kit (AKOYA Biosciences, Marlborough, MA, USA), following the manufacturer’s instructions. Briefly, slides were baked for 60 min at 58 °C. Slides were then dewaxed sequentially in xylene twice for 5 min, 100% ethanol three times for 1 min, 70% ethanol for 1 min and then distilled water on the Gemini AS automated slide scanner (Epredia, Kalamazoo, MI, USA) on the auto Stainer program, DEWAX. The slides were washed in distilled water for 10 min. Heat-induced epitope retrieval (HIER) was performed at 110 °C for 5 min using the Decloaking chamber TM NxGen (Biocare Medical, Pacheco, CA, USA) in citrate buffer pH 6 (DAKO). Slides were washed in distilled water, followed by Tris Buffered Saline (TBS) (DAKO, Glostrup, Denmark). Slides were then incubated in 0.1 M glycine in a TBS block (DAKO, Denmark), washed in distilled water and then in TBS. The following steps were then completed on the Leica Bond RXm automated immunostainer (Leica Biosystems, Germany) following the BOND protocol. The steps were completed sequentially five times to apply the following antibodies; rabbit anti-ionised calcium-binding adapter molecule 1 (IBA-1, WAKO 019-19741 1:4000), anti-glial fibrillary acidic protein (GFAP, Abcam ab7260 1:3000), anti-CD163 (Abcam ab182422 1:1500), anti-S100A10 (ThermoFisher PA5-95505 1:2000), anti-P2RY12 (AnaSpec AS-55043A 1:1000). After the 5th round of primary antibody application, the Opal Polaris 780 reagent pack (AKOYA Biosciences, USA) was used to complete the final steps. OPAL tyramide signal amplification DIG working solution was added at a concentration of 1:150 and incubated for 10 min at RT. The solution was removed, 780 Polaris fluorophore was added at a concentration of 1:25 and left to incubate for 60 min at RT (IBA-1-480, GFAP-520, CD163-570, S100A10-690 and P2RY12-780). Slides were washed in TBS 3 times for 2 min, once in distilled water and once more with TBS. Then, a 4′,6-diamidino-2-phenylindole (DAPI) working solution was added and left to incubate for 5 min at RT. Slides were washed in TBS for 2 min and then with distilled water for 2 min. Finally, the slides were cover-slipped with Prolong Gold Antifade Mounting Medium (ThermoFisher, USA). Slides were imaged on Vectra Polaris (PerkinElmer, Shelton, CT, USA), an automated imaging system, at 20× objective.

### 2.10. Immunohistochemistry

Sections were washed with PBS before HIER performed at 95 °C for 5 min using the Decloaking chamber TM NxGen (Biocare Medical, USA) in citrate buffer pH 6 (DAKO). After that, slides were washed in PBS and treated with 0.1% sodium borohydride in PBS. Slides were then incubated in PBS with 0.3% Triton-X (Sigma-Aldrich, Saint Louis, MO, USA) for 30 min at RT, then replaced by a blocking solution containing PBS with 10% normal donkey serum (NDS) and 0.3% Triton X-100 (both from Sigma-Aldrich) for another 30 min at RT. Sections were incubated with the following primary antibodies diluted in PBS containing 2% NDS and 0.1% Triton X-100 overnight at 4 °C; goat anti-mouse IBA-1 (WAKO 011-27991 1:200), rabbit anti-mouse lysozyme (LYZ, Abcam ab108508 1:200), goat anti-mouse CD206 (RnDSystems AF2535 1:400), rabbit anti-P2RY12 (AnaSpec AS-55043A 1:400), rabbit anti-GFAP (Abcam ab7260 1:400), goat anti-C3 (Abcam ab11887 1:100), hamster anti-mouse CD11c (ThermoFisher 14-0114-82 1:100) and rabbit anti-mouse insulin growth-like factor 1 (IGF1, Abcam ab9572 1:100). Sections were then washed three times with PBS before adding the following suitable secondary antibodies: Alexa Fluor 488 donkey anti-rabbit/goat/hamster and Alexa Fluor 647 donkey anti-rabbit/goat (1:500; Life Technologies) in PBS with 2% NDS. The slides were incubated at RT for 2 h. Finally, sections were washed five times with PBS, and Prolong Gold Antifade Reagent with DAPI (Life Technologies) was applied before slides were coverslipped and stored at 4 °C until imaging. All images were acquired using the Zeiss LSM 900 confocal microscope with 20× objective at 512 × 512-pixel resolution. The spinal cord sections were imaged as a whole using the tile scan option with a 6 µm Z stack taken.

### 2.11. Image Analysis

Opal Multiplex immunohistochemistry images were first traced, and selected tissues were unmixed in Phenochart (AKOYA Biosciences, USA). For all staining, the ipsilateral (left) dorsal and ventral horns were identified, and the area of positive staining quantified for IBA-1, P2RY12, GFAP, S100A10, LYZ and CD206 via thresholding in either HALO (Indica Labs, Albuquerque, NM, USA) or ImageJ (Bethesda, MD, USA). Each image was thresholded to remove the background and converted to an 8-bit grey-scale image. The area of pixels above the threshold was measured as a percentage area of the region of interest. For GFAP and S100A10 colocalisation, the object colocalisation module in HALO (Indica labs, USA) was used, whilst CD163+, GFAP+C3+ and CD11c+IGF1+ cells were counted manually using the cell counter plugin in ImageJ (Bethesda, USA). The experimenter was blinded to groups during analysis.

### 2.12. Statistical Analysis

The estimation approach to inference that emphasises reporting effect sizes with interval estimates rather than the approach of null-hypothesis significance testing was used [36]. Data from behaviour, flow cytometry, and immunohistochemistry were analysed with multiple two groups on the open-source platform Estimation Statistics (estimationstats.com, last accessed 21 June 2023). The effect size was calculated using Hedge’s g to compare the means of saline-based vehicle (D-PBS) treated versus Tregs treated groups and sham surgery versus CCI groups. Effect sizes are categorised into small (g < 0.5), medium (0.5 < g < 0.8), large (0.8 < g < 1.2), and very large (g > 1.2). The 95% confidence interval (CI) of the mean difference was calculated by performing bootstrap resampling on 5000 samples. Emphasis was given to effect sizes and their CI, and effect sizes categorised as large (* 0.8 < g < 1.2) and very large (** g > 1.2) were reported along with the *p*-value. Graphs were created on GraphPad Prism 9 software (GraphPad Inc., San Diego, CA, USA).

### 2.13. Data Availability

The authors agree to make all raw data available. Furthermore, the raw data of this study will be made available on request to G.M.-T. (gila@unsw.edu.au).

## 3. Results

### 3.1. Activated Regulatory T Cells Migrate to and Drain from the Meninges into the Peripheral Lymphatic System 48 h following Intrathecal Delivery in Naïve Mice

**Figure 1:**
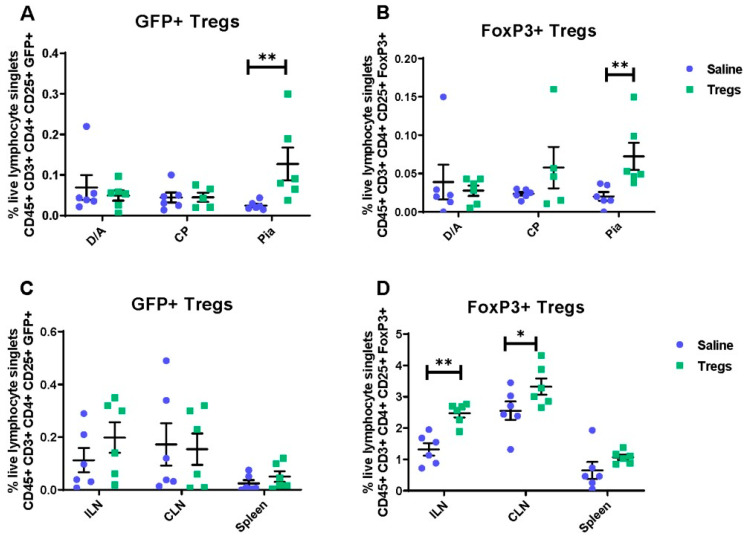
Adoptively transferred Tregs accumulate in the pia 48 h after intrathecal injection in naïve mice. (**A**) GFP+ Tregs, or (**B**) FoxP3+ Tregs in the meninges and choroid plexus and (**C**) GFP+ Tregs, or (**D**) FoxP3+ Tregs in the peripheral lymphatic system of naïve female mice 48 h post-intrathecal delivery of activated Tregs, or saline-based vehicle (control). Raw data was analysed using estimation statistics (* = large effect size, 0.8 < g < 1.2; ** = very large effect size, g > 1.2 for Treg-recipient mice compared to saline group, *n* = 5–6 per group). D/A = dura/arachnoid, CP = choroid plexus. Data expressed as mean ± SEM.

To investigate the effects of Tregs on neuropathic pain, peripheral immunity, and spinal cord gliosis, we used intrathecal delivery of GFP+ Tregs. Because this is a route of administration via an injection into the subarachnoid space, we first determined if Tregs can migrate through the meninges and choroid plexus and enter the peripheral lymphatic system in naïve mice. To this end, activated Tregs were delivered intrathecally into naïve female C57BL/6J recipient mice, with vehicle control animals receiving intrathecal D-PBS (saline group), and 48 h later, mice were culled, and the meninges, choroid plexus, and peripheral lymphatic tissue harvested for flow cytometric analysis of both FoxP3 (a marker of Tregs) and GFP. FoxP3 analysis was incorporated to assess endogenous Tregs and adoptively transferred GFP+ Tregs. In the CNS borders, an increase in GFP+ Tregs was seen in the pia mater of Tregs-recipient mice (g = 1.34 [95.0%CI 0.733, 1.89], *p* = 0.002) compared to vehicle-treated animals (Figure 1A). Similarly, when identified through FoxP3 expression (Figure 1B), this increase in Tregs was also observed (g = 1.49 [95.0%CI 0.893, 2.02], *p* < 0.0001) compared to mice that received intrathecal vehicle injection. In the peripheral lymphatic system, when identified through GFP, no differences in GFP+ Tregs were seen in any measured tissue (Figure 1C). However, FoxP3+ Tregs were increased in the inguinal and cervical LNs (Figure 1D) following intrathecal delivery of activated Tregs compared to vehicle (inguinal LNs: g = 2.57 [95.0%CI 1.23, 4.04], *p* = 0.0024; cervical LNs: g = 1.04 [95.0%CI −0.199, 1.93], *p* = 0.0782), suggesting adoptive transfer of GFP+ Tregs may expand the endogenous Treg population.

### 3.2. Spinal Delivery of Activated Tregs Reduces Mechanical Allodynia and Improves Exploratory Behaviours in Both Male and Female Mice following Peripheral Nerve Injury

**Figure 2:**
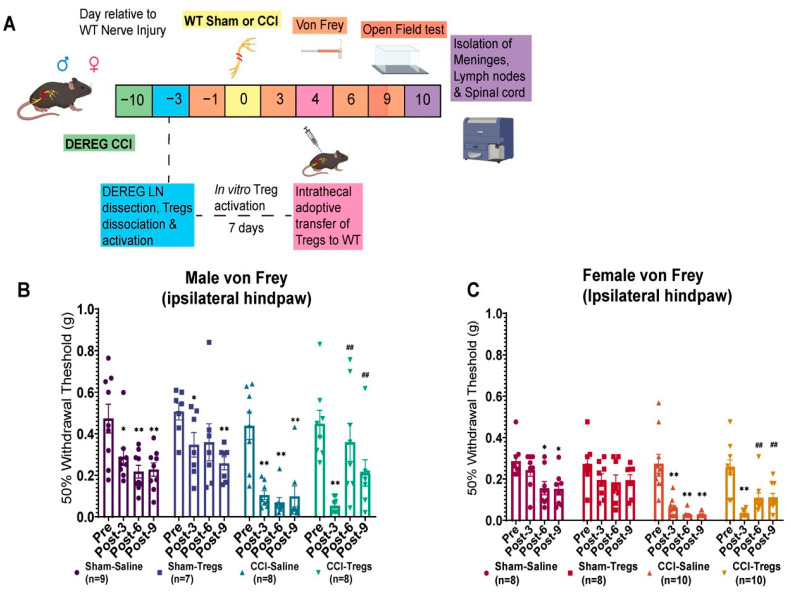
Adoptive transfer using intrathecal administration of Tregs reduces pain behaviours following CCI. (**A**) Schematic of experimental timeline for adoptive transfer of Tregs following CCI, behavioural assays, and tissue dissection. Nerve-injured and sham-operated mice were injected with activated Tregs or saline-based vehicle (control). Mechanical allodynia in CCI is reduced in males (**B**) and females (**C**) following intrathecal delivery of activated Tregs. Mice developed reduced paw withdrawal thresholds following CCI (* 0.8 < g < 1.2 and ** g > 1.2 relative to pre-injury), which transiently recovered following spinal delivery of Tregs (## = relative to day 3, pre-Treg injection, *n* = 7–10 per group).

Treatment with Tregs reduces neuropathic pain behaviours in males and females [12,14,16], and Treg expansion within the spinal cord meninges inhibits microglia-induced pain hypersensitivity in female mice only [27]. Thus, we compared the effects of intrathecal delivery of Tregs on pain behaviours after peripheral nerve injury in male and female mice. C57BL/6J mice underwent CCI as described, and four days following this procedure, mice received an intrathecal injection of either activated Tregs or vehicle (Figure 2A). Mechanical allodynia was assessed using calibrated von Frey filaments before surgery (baseline) and on days 3, 6, and 9 post-injury, while exploratory behaviours were evaluated using an open field apparatus on day 9 (Figure 2A). Both male and female mice developed mechanical allodynia post-CCI, confirmed by a reduced paw withdrawal threshold following CCI surgery of the sciatic nerve (Figure 2B,C). Intrathecal delivery of activated Tregs attenuated this pain hypersensitivity in both sexes, increasing mechanical withdrawal thresholds on day 6 (Figure 2B, males: g = 1.56 [95.0%CI 0.722, 2.32), *p* = 0.002; Figure 2C, females: g = 1.46 [95.0%CI 0.891, 2.18], *p* = 0.001) and day 9 (males: g = 1.51 [95.0%CI 0.211, 1.73], *p* = 0.001; females: g = 1.87 [95.0%CI 1.29, 2.45], *p* = 0.001) following CCI, relative to pre-Treg injection (day three after CCI).

**Figure 3:**
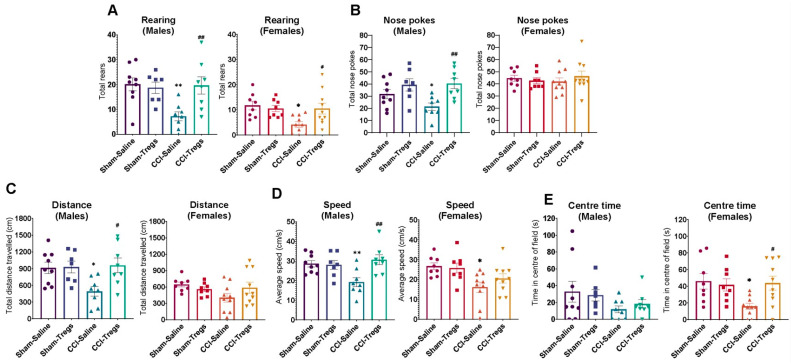
Intrathecal administration of activated Tregs improves exploratory behaviours in male and female mice following CCI. Nerve-injured and sham-operated mice were injected with activated Tregs or saline-based vehicle (control). The open field hole-board test was utilised to measure the number of rears (**A**), nose pokes (**B**), distance covered (**C**), average speed (**D**), as well as time spent in the centre of the open field (**E**). Testing was conducted on the 9th day after CCI. Data are shown as mean ± SEM (* 0.8 < g < 1.2 and ** g > 1.2 for CCI relative to sham saline and # 0.8 < g < 1.2 and ## g > 1.2 for Tregs relative to CCI saline control, *n* = 7–10 per group).

Similarly, both male and female mice showed a reduction in exploratory behaviours (Figure 3), as well as increased anxiety-like behaviour (limited time spent in the centre of the open field apparatus) in female mice (Figure 3E) following CCI. In males, Treg injection following CCI resulted in increased rearing (Figure 3A, g = 1.5 [95.0%CI 0.488, 2.43], *p* = 0.005), nose pokes (Figure 3B, g = 1.72 [95.0%CI 0.8, 2.71], *p* = 0.0026), distance travelled (Figure 3C, g = 1.41 [95.0%CI 0.352, 2.31], *p* = 0.0114), and speed (Figure 3D, g = 1.57 [95.0%CI 0.448, 2.52], p 0.0058) compared to CCI mice which received intrathecal vehicle. In females, spinal delivery of activated Tregs post-CCI increased rearing (Figure 3A, g = 1.13 [95.0%CI 0.361, 1.86], *p* = 0.012), and time spent in the centre of the apparatus (reduced anxiety-like behaviour) (Figure 3E, g = 1.3 [95.0%CI 0.321, 2.28], *p* = 0.0084) compared to the CCI saline group.

### 3.3. Activated Tregs Migrate from the Meninges into Peripheral Lymph Nodes 6 Days following Spinal Delivery in Both Control and Nerve-Injured Mice

Given intrathecal delivery of activated Tregs can reduce pain behaviours in male and female mice following CCI, we aimed to determine if nerve injury influenced the migration of Tregs from the meninges into the peripheral lymphatic system. To determine where adoptively transferred Tregs are located following CCI, activated GFP+ Tregs were delivered intrathecally into naïve or nerve-injured male and female C57BL/6J recipient mice, with control animals receiving intrathecal D-PBS (saline group). Mice were culled six days following intrathecal Treg delivery, and the meninges, choroid plexus, and peripheral lymph nodes were harvested for flow cytometric analysis of GFP+ Tregs.

**Figure 4:**
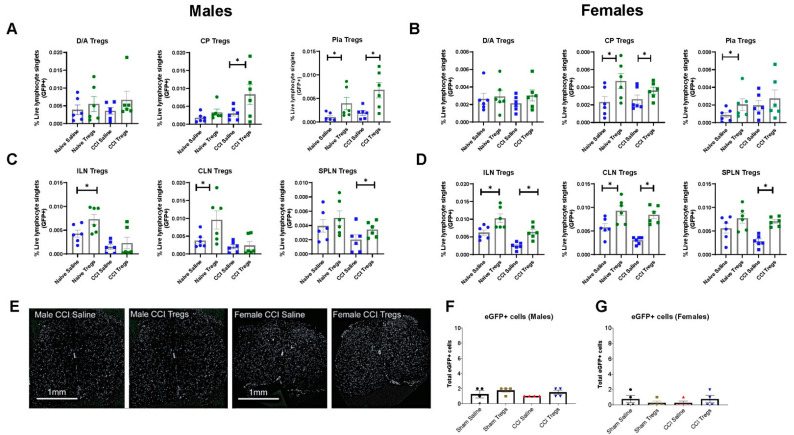
Tracking intrathecally injected GFP+ Tregs following CCI. GFP+ cells in the meninges and choroid plexus of male (**A**) and female (**B**) mice, as well as in the peripheral lymphatics of male (**C**) and female (**D**) mice following intrathecal delivery of activated Tregs or saline-based vehicle (control) in naïve and nerve-injured mice. (* 0.8 < g < 1.2 for Tregs relative to saline control, *n* = 6 per group). D/A = dura/arachnoid, CP = choroid plexus, ILN = inguinal lymph nodes, CLN = cervical lymph nodes, SPLN = sciatic/popliteal lymph nodes. (**E**) Representative images taken from the lumbar spinal cord stained for eGFP+ cells (green) counterstained with DAPI (blue/white specks) in nerve-injured male and female mice following vehicle and GFP+ Treg injection. No eGFP+ cells were identified in the spinal cord, with total eGFP+ cell numbers shown in male (**F**) and female (**G**) mice following intrathecal delivery of activated Tregs or vehicle in naïve and nerve-injured mice (*n* = 4 per group).

In the meninges and choroid plexus (Figure 4A,B) of naïve male and female mice, an increase in GFP+ Tregs was seen in the pia mater of Tregs-recipient mice (male g = 1.13 [95.0%CI 0.42, 2.01], *p* = 0.0232 and female g = 0.901 [95.0%CI −0.288, 1.74], *p* = 0.098), and in the choroid plexus in female Tregs-recipient mice (g = 1.18 [95.0%CI 0.062, 2.52], *p* = 0.0408) compared to vehicle-treated animals. In the peripheral lymphatic system, differences in GFP+ Tregs 6 days following intrathecal delivery of activated Tregs were seen in the inguinal LNs (male g = 1.22 [95.0%CI 0.177, 2.64], *p* = 0.0224 and female g = 1.48 [95.0%CI 0.648, 2.46], *p* = 0.0224) and cervical LNs (male g = 1.14 [95.0%CI 0.0769, 2.37], *p* = 0.0638 and female g = 1.43 [95.0%CI −0.298, 2.68], *p* = 0.0272). In nerve-injured male and female mice, an increase in GFP+ Tregs was observed in the choroid plexus (male g = 0.999 [95.0%CI −0.504, 2.04], *p* = 0.0808 and female g = 0.828 [95.0%CI −0.401, 2.57], *p* = 0.146), and in the pia in male Tregs-recipient mice (g = 1.53 [95.0%CI 0.308, 2.77], *p* = 0.019) compared to vehicle-treated mice. In the peripheral lymphatic system (Figure 4C,D), differences in GFP+ Tregs 6 days following CCI were seen in the sciatic/popliteal LNs in both sexes (male g = 0.916 [95.0%CI −0.644, 2.3], *p* = 0.125 and female g = 3.45 [95.0%CI 2.1, 4.78], *p* = 0.0002) and the inguinal and cervical LNs in female mice (inguinal g = 2.37 [95.0%CI 1.18, 3.54], *p* < 0.0001 and cervical g = 3.5 [95.0%CI 2.67, 4.59], *p* = 0.0002). There was no infiltration of GFP+ Tregs into the lumbar spinal cord parenchyma of male or female mice, at least on day six following spinal intrathecal delivery of Tregs (Figure 4E–G).

### 3.4. Spinal Delivery of Activated Tregs Induces Differential Changes in Immune Cell Profile in the Meninges and Choroid Plexus of Male and Female Mice following Nerve Injury

Meningeal inflammation is well-established in chronic autoimmune CNS lesions, such as multiple sclerosis and EAE [37]. Since Tregs are potently anti-inflammatory, and inflammation is essential to the onset and progression of EAE, we first sought to investigate whether the intrathecal delivery of activated Tregs could ameliorate inflammation in the meninges and choroid plexus and affect disease progression. EAE was induced in 9-week-old female DEREG donor mice, and GFP+ Tregs were isolated 30 days post-induction and activated in vitro for seven days. Tregs were then delivered intrathecally into EAE-affected C57BL/6J female mice on day 12 following the onset of the disease. Control groups included CFA-injected mice, which received activated Tregs intrathecally, and EAE-affected mice, which received intrathecal vehicle injection. Tissue was harvested on day 16 (i.e., the clinical peak of disease) and analysed for immune cell profile by flow cytometry (Figure S2). Mice developed typical EAE with symptom onset between 9–11 days post-induction, a mean clinical score of 3.5 on day 16 (Figure S2C), and symptom severity corresponding to increased loss in body weight (Figure S2D). Control CFA mice did not develop clinical symptoms over the 16-day monitoring period. No effect of intrathecal injection of Tregs (following disease onset) on clinical EAE or body weight was observed (Figure S2C,D) in line with our previous study [12]. Flow cytometry of the meninges and choroid plexus 16 days post-EAE induction revealed many immunological changes between control mice and both EAE groups, as well as changes between the EAE groups, injected intrathecally with either vehicle or activated Tregs, summarised in Figure S2A. In particular, increases in both EAE groups compared to the control group were seen in total leukocytes, T cells, B cells, dendritic cells, and monocytes/macrophages in both the dura/arachnoid and the pia, with fewer changes in the choroid plexus. Treg treatment induced decreases in the proportions of both T helper cell and cytotoxic T cell populations in the dura/arachnoid, T helper cells and CD11b− DCs in the choroid plexus, and CD45+ leukocytes, T helper cells, FoxP3+ Tregs, and CD11b− DCs in the pia in EAE + Tregs mice compared to EAE + vehicle animals. Additionally, Treg treatment-induced increases in the proportion of monocytes/macrophages in the choroid plexus and GFP+ Tregs in the pia in EAE + Tregs mice compared to EAE + vehicle animals (Figure S2A).

**Figure 5:**
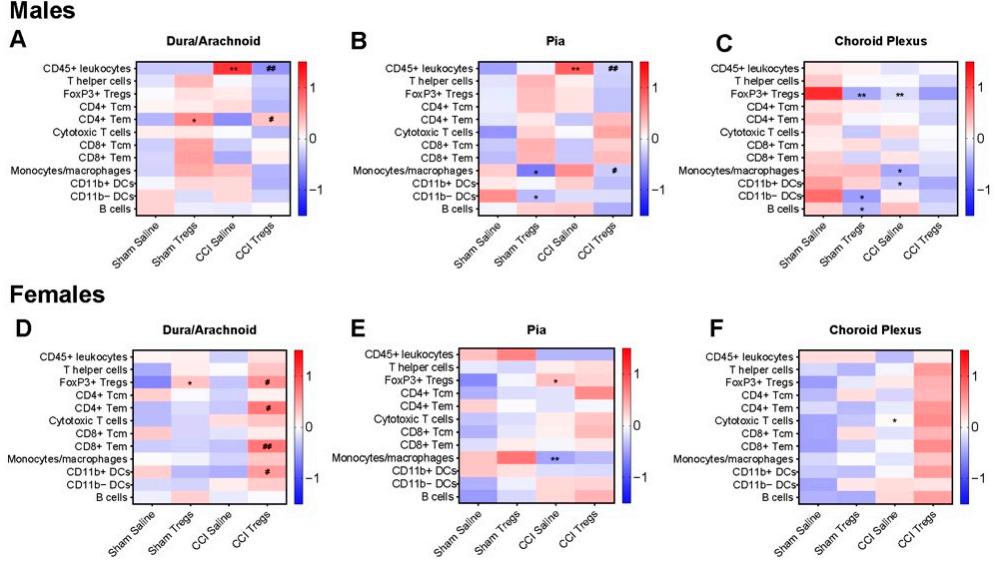
Immunological changes in the meninges and choroid plexus following intrathecal Treg injection in nerve-injured male and female mice. Heatmaps summarising immunological changes in the (**A**) dura/arachnoid, (**B**) pia, and (**C**) choroid plexus of male mice and (**D**) dura/arachnoid, (**E**) pia, and (**F**) choroid plexus of female mice following peripheral nerve injury or sham surgery and intrathecal delivery of activated Tregs, or saline-based vehicle (control). Data normalised for the heatmaps. Raw data were analysed using estimation statistics. (* 0.8 < g < 1.2 and ** g > 1.2 for differences relative to sham saline and # 0.8 < g < 1.2 and ## g > 1.2 relative to CCI saline, *n* = 7–10 per group).

We next investigated whether the immune cell profile of the meninges and choroid plexus is altered in the context of CCI of the sciatic nerve and CCI following treatment with activated Tregs in male and female mice. As above, 8-week-old male and female C57BL/6J mice underwent CCI, and four days following this procedure, mice received an intrathecal injection of either activated Tregs or vehicle (Figure 2A). 10 days post-surgery, mice were euthanised, and meninges and choroid plexus tissues were isolated for flow cytometric analysis. In the meninges of male mice, numerous changes were observed relative to the sham + vehicle control group, summarised in Figure 5A–C. In the dura/arachnoid (Figure 5A), the proportion of CD45+ leukocytes was increased following CCI in vehicle-treated mice compared to sham injury (g = 1.48 [95.0%CI 0.61, 2.23], *p* = 0.0032). There was an increase in CD4+ Tem cells observed following Treg injection in sham (g = 1.15 [95.0%CI 0.214, 2.23], *p* = 0.0296) and CCI-injured (g = 0.838 [95.0%CI −0.0367, 1.66], *p* = 0.129) mice. A reduction in CD45+ leukocytes was also seen in CCI mice after Treg injection compared to vehicle-treated mice (g = −1.51 [95.0%CI −2.36, −0.661], *p* = 0.0058). The pia mater (Figure 5B) showed an increased proportion of CD45+ leukocytes (g = 1.51 [95.0%CI 0.464, 2.36], *p* = 0.0056) in CCI mice compared to the sham injury group after vehicle treatment, as well as a reduction in CD11b− DCs (g = −0.873 [95.0%CI −2.02, 0.189], *p* = 0.098), and monocytes/macrophages (g = −1.03 [95.0%CI −1.99, −0.118], *p* = 0.0474) following Treg treatment in sham injured mice. Further, Treg treatment following CCI also reduced total CD45+ leukocytes (g = −1.2 [95.0%CI −2.11, −0.19], *p* = 0.0234) and monocytes/macrophages (g = −0.862 [95.0%CI −1.97, 0.303], *p* = 0.081). In the choroid plexus (Figure 5C), CCI-injured male mice displayed reduced FoxP3+ Tregs (g = −1.37 [95.0%CI −2.26, −0.3], *p* = 0.027), CD11b+ DCs (g = −0.821 [95.0%CI −2.17, 0.559], *p* = 0.143), and monocytes/macrophages (g = −0.912 [95.0%CI −2.39, 0.441], *p* = 0.121) compared to sham injury. In sham mice, treatment with Tregs reduced FoxP3+ Tregs (g = −1.61 [95.0%CI −2.68, −0.565], *p* = 0.0096), B cells (g = −1.07 [95.0%CI −2.71, 0.394], *p* = 0.0674), and CD11b− DCs (g = −1.25 [95.0%CI −3.04, 0.209], *p* = 0.0404) compared to vehicle-treated mice. 

The immune cell profile in female mice that had undergone CCI and intrathecal delivery of either activated Tregs or vehicle was also significantly altered in the meninges (summarised in Figure 5D–F), although distinct from the changes seen in male mice. In the dura/arachnoid mater of female mice (Figure 5D), increases were seen in FoxP3+ Tregs after Treg treatment in sham (g = 1.08 [95.0%CI −0.0708, 2.22], *p* = 0.0432) and CCI mice (g = 0.976 [95.0%CI 0.0577, 1.84], *p* = 0.0386). There was also an increase in CD11b+ DCs (g = 1.1 [95.0%CI 0.0742, 2.15], *p* = 0.0182), CD4+ Tem cells (g = 0.941 [95.0%CI −0.00198, 1.61], *p* = 0.0288) and CD8a+ Tem cells (g = 1.21 [95.0%CI 0.361, 2.0], *p* = 0.0102) in CCI mice injected with Tregs compared to vehicle controls. Changes in the pia and choroid plexus (Figure 5E,F) of female mice were less than in other analysed tissue, with only an increase in cytotoxic T cells (g = 0.898 [95.0%CI 0.0284, 1.7], *p* = 0.0664) observed in the choroid plexus of CCI injured mice compared to sham control mice (Figure 5F). In the pia mater of female nerve-injured mice (Figure 5E), there was an elevation in FoxP3+ Tregs (g = 0.923 [95.0%CI 0.2, 1.49], *p* = 0.0454) and a decrease in monocytes/macrophages (g = −1.33 [95.0%CI −2.82, −0.156], *p* = 0.0072).

### 3.5. Spinal Delivery of Activated Tregs Induces Differential Changes in Immune Cell Profile in the Peripheral Lymphatic Tissue of Male and Female Mice following Nerve Injury

Since Tregs were shown to traffic out of the meninges to the peripheral lymphatic tissues following intrathecal administration (Figure 1 and Figure 4), we examined their effects on the immune cell profile in peripheral lymph nodes and the spleen by flow cytometry. Changes in the immune profile of peripheral lymphatic tissues in the EAE-affected female C57BL/6J mice (16 days post-immunisation) treated with intrathecal delivery of activated Tregs or vehicle control (12 days post-immunisation) are summarised in Figure S2B. Increases in GFP+ Tregs and monocytes/macrophages in the inguinal LNs and increases in GFP+ Tregs and CD11b+ DCs in the cervical LNs were seen in EAE + Tregs mice compared to EAE + vehicle animals. In the spleen, T helper cells were increased, while CD45+ leukocytes, CD11b− DCs, and B cells were decreased in EAE + Tregs mice compared to EAE + vehicle groups (Figure S2B).

**Figure 6:**
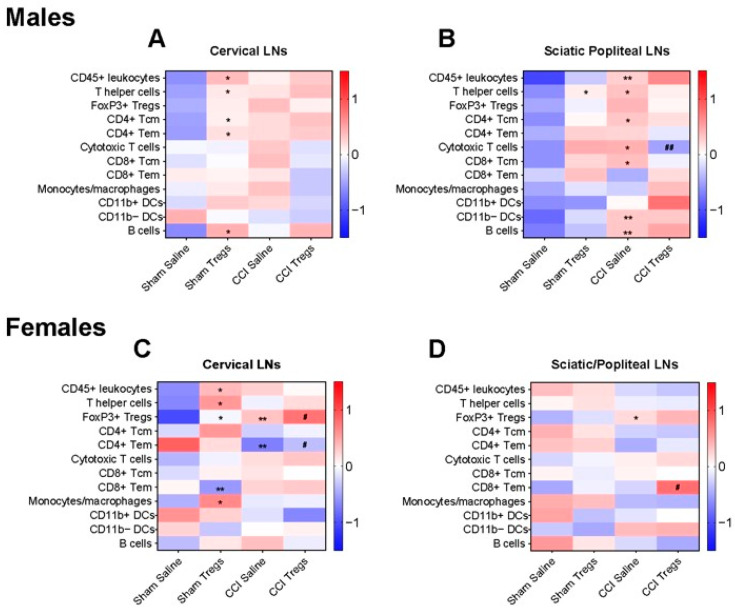
Immunological changes in peripheral lymph nodes following intrathecal Treg injection in nerve-injured male and female mice. Heatmaps summarising immunological changes in the (**A**) cervical LNs and (**B**) sciatic/popliteal LNs of male mice, and cervical LNs (**C**) and sciatic/popliteal LNs (**D**) of female mice following peripheral nerve injury or sham surgery and intrathecal delivery of activated Tregs, or saline-based vehicle (control). Data normalised for the heatmaps. Raw data were analysed using estimation statistics. (* 0.8 < g < 1.2 and ** g > 1.2 for differences relative to sham saline and # 0.8 < g < 1.2 and ## g > 1.2 relative to CCI saline, *n* = 7–10 per group).

Numerous changes in immune cell profile were also found in the peripheral lymphatic tissues of mice with peripheral nerve injury following treatment with intrathecal delivery of activated Tregs (Figure 6 and Figure S3).

In males, in the cervical LNs (Figure 6A), many cell populations were increased following Treg treatment compared to vehicle control in sham mice, including CD45+ leukocytes (g = 0.862 [95.0%CI −0.382, 2.01], *p* = 0.0904), T helper cells (g = 0.978 [95.0%CI 0.0911, 1.88], *p* = 0.0438), B cells (g = 0.963 [95.0%CI −0.277, 2.23], *p* = 0.0608), CD4+ Tem cells (g = 0.932 [95.0%CI −0.0758, 2.06], *p* = 0.0658), and CD4+ Tcm cells (g = 0.945 [95.0%CI −0.0098, 1.91], *p* = 0.059).

In the sciatic/popliteal LNs (Figure 6B), the LNs closest to the sciatic nerve injury site, many changes were also observed in males. An increase in several immune cell populations was found following CCI, with CD45+ leukocytes (g = 2.4 [95.0%CI 0.418, 7.8], *p* = 0.0032), T helper cells (g = 1.16 [95.0%CI 0.505, 2.09], *p* = 0.0198), cytotoxic T cells (g = 1.06 [95.0%CI 0.153, 2.14], *p* = 0.0638), B cells (g = 1.44 [95.0%CI −0.0612, 3.31], *p* = 0.0172), CD11b− DCs (g = 1.3 [95.0%CI 0.175, 2.54], *p* = 0.0244), CD11b+ DCs (g = 0.909 [95.0%CI −0.23, 2.09], *p* = 0.09), CD4+ Tcm cells (g = 1.06 [95.0%CI 0.418, 1.88], *p* = 0.0266), and CD8a+ Tcm cells (g = 0.932 [95.0%CI −0.0387, 1.52], *p* = 0.0426) all elevated compared to the sham + vehicle mice. Treg injection increased T helper cells (g = 0.962 [95.0%CI 0.962, 1.59], *p* = 0.061) in sham mice and reduced cytotoxic T cells in nerve-injured mice (g = −1.26 [95.0%CI −2.34, −0.355], *p* = 0.0172).

In the inguinal LNs of males, Treg injection increased both CD11b− DCs and CD11b+ DCs in CCI-injured mice compared to vehicle-treated animals (Figure S3A). In the spleen of males, cytotoxic T cells and monocytes/macrophages were decreased in CCI + Tregs animals compared to CCI + vehicle (Figure S3B).

In females, in the cervical LNs (Figure 6C), Treg treatment in sham injured mice caused increases in CD45+ leukocytes (g = 0.939 [95.0%CI −0.329, 1.7], *p* = 0.0842), T helper cells (g = 1.4 [95.0%CI −0.256, 2.79], *p* = 0.0286), FoxP3+ Tregs (g = 0.843 [95.0%CI −0.41, 2.54], *p* = 0.142), and monocytes/macrophages (g = 1.07 [95.0%CI −0.0478, 2.41], *p* = 0.0746), whilst CD8a+ Tem cells decreased (g = −1.22 [95.0%CI −1.72, −0.467], *p* = 0.0088) compared to vehicle-treated mice. There was also a decrease in CCI-injured control mice for CD4+ Tem cells (g = −1.5 [95.0%CI −2.97, 0.0841], *p* = 0.0154), whilst FoxP3+ Tregs were elevated in the cervical LNs (g = 1.88 [95.0%CI 0.522, 6.15], *p* = 0.0112) compared to sham mice. Treg treatment in CCI mice caused an increase in FoxP3+ Tregs (g = 0.982 [95.0%CI −0.219, 2.02], *p* = 0.124) and CD4+ Tem cells (g = 0.864 [95.0%CI −0.434, 2.16], *p* = 0.124) relative to vehicle control mice.

Changes in the sciatic/popliteal LNs in females (Figure 6D) were limited, with nerve-injured mice displaying an increase in FoxP3+ Tregs (g = 1.04 [95.0%CI 0.25, 1.89], *p* = 0.0378), and CCI Treg-treated mice having elevated CD8a+ Tem cells compared to the saline vehicle group (g = 1.12 [95.0%CI 0.203, 2.14], *p* = 0.0208).

In the inguinal LNs of females (Figure S3C), CCI induced several changes in cytotoxic T cells, FoxP3+ Tregs, CD11b+ DCs and CD4+ Tem cells relative to sham mice. Following Treg injection in sham mice, an increase was seen in T helper cells and monocytes/macrophages relative to saline control mice, along with a decrease in CD11b− DCs and CD8a+ Tcm cells. In the spleen (Figure S3D), numerous changes were observed relative to sham saline control animals. Treg treatment increased T helper cells, FoxP3+ Tregs, CD11b− DCs, and monocytes/macrophages in sham-injured mice. Similarly, when compared to sham vehicle mice, CCI increased T helper cells, cytotoxic T cells, FoxP3+ Tregs, and CD8a+ Tcm cells.

### 3.6. Treatment with Tregs Reduces Spinal Microglial Reactivity and Induces an Anti-Inflammatory Shift in Nerve-Injured Male, but Not Female, Mice

**Figure 7:**
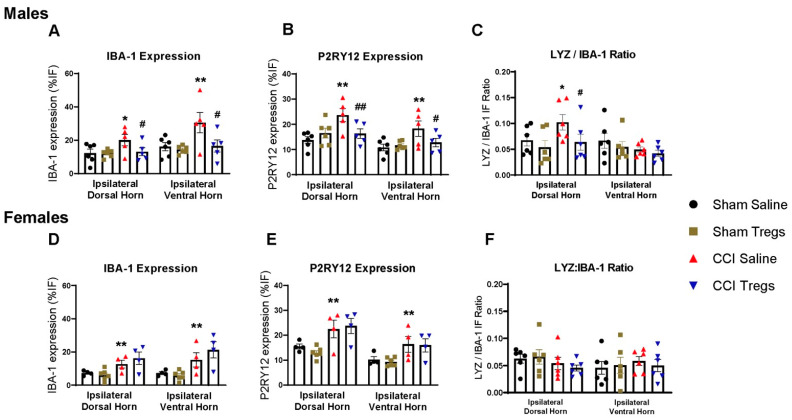
Changes in microglial phenotype in the ipsilateral spinal cord following intrathecal administration of Tregs in male and female mice. Nerve-injured and sham-operated mice were injected with activated Tregs or saline-based vehicles (control). Opal multiplex immunohistochemistry and immunofluorescent staining were used to assess microglial markers (**A**) IBA-1, (**B**) P2RY12, (**C**) LYZ:IBA-1 ratio in males and (**D**) IBA-1, (**E**) P2RY12, (**F**) LYZ:IBA-1 ratio in females in the ipsilateral dorsal and ventral horns. Data are shown as mean ± SEM (* 0.8 < g < 1.2 and ** g > 1.2 for changes relative to sham saline and # 0.8 < g < 1.2 and ## g > 1.2 relative to CCI saline, *n* = 4–6 per group).

**Figure 8:**
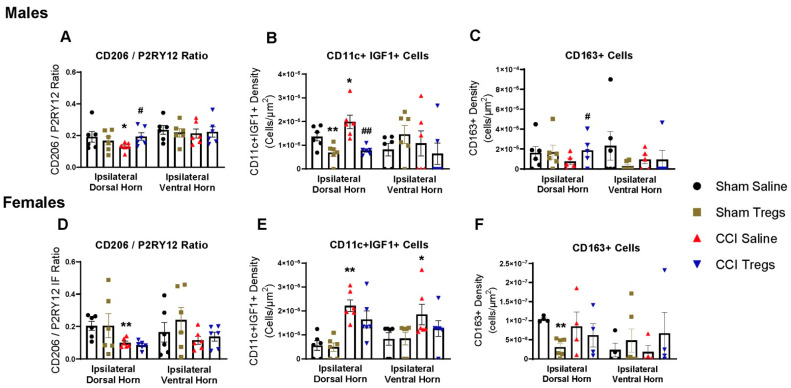
Changes in macrophage/microglial phenotype in the ipsilateral spinal cord following intrathecal administration of Tregs in male and female mice. Nerve-injured and sham-operated mice were injected with activated Tregs or saline-based vehicles (control). Opal multiplex immunohistochemistry and immunofluorescent staining were used to assess macrophage/microglial markers (**A**) CD206: P2RY12 ratio, (**B**) CD11c+ IGF1+ cell density and (**C**) CD163+ cell density in males and (**D**) CD206: P2RY12 ratio, (**E**) CD11c+ IGF1+ cell density and (**F**) CD163+ cell density in females in the ipsilateral dorsal and ventral horns. Data are shown as mean ± SEM (* 0.8 < g < 1.2 and ** g > 1.2 for changes relative to sham saline and # 0.8 < g < 1.2 and ## g > 1.2 relative to CCI saline, *n* = 4–6 per group).

To evaluate spinal macrophage/microglial responses following peripheral nerve injury and Treg treatment, lumbar spinal sections from male and female mice were immunohistochemically stained using microglial reactivity markers (IBA-1 and P2RY12), as well as inflammatory (LYZ) (Figure 7), anti-inflammatory (CD206) and pain-resolving (CD11c and IGF-1; CD163) macrophage/microglial phenotypic markers (Figure 8). Representative images are illustrated in Figures S4–S9.

In males, nerve-injured mice exhibited robust microglia reactivity in both the ipsilateral dorsal (IBA-1 g = 1.08 [95.0%CI −0.506, 2.46], *p* = 0.103 Figure 7A and P2RY12 g = 1.99 [95.0%CI 0.508, 3.65], *p* = 0.0056 Figure 7B) and ventral (IBA-1 g = 1.3 [95.0%CI −1.15, 2.74], *p* = 0.0338 and P2RY12 g = 1.35 [95.0%CI 0.0729, 3.68], *p* = 0.0458) horns compared to sham-injured mice. There was also a shift to inflammatory microglia, indicated by an increase in LYZ expression relative to IBA-1 (g = 1.0 [95.0%CI −0.0762, 2.1], *p* = 0.0872 Figure 7C) and a decrease in CD206 expression relative to P2RY12 (g = −0.921 [95.0%CI −1.66, 0.31], *p* = 0.095 Figure 8A) in the ipsilateral dorsal horn. Although extremely few CD11c+IGF1+ cells were present in the spinal cord (day ten post-injury), CCI also increased the density of pain-resolving microglia (g = 0.974 [95.0%CI −0.155, 1.79], *p* = 0.095 Figure 8B). CD163 cell density in the spinal cord parenchyma was low, given they are primarily located in the meninges and vasculature [38], and their density was not changed following CCI (Figure 8C). Noticeably, treatment with Tregs attenuated the increase in microglia reactivity in both the ipsilateral dorsal (IBA-1 g = −0.957 [95.0%CI −2.83, 0.546], *p* = 0.108 and P2RY12 g = −1.31 [95.0%CI −2.81, 0.174], *p* = 0.042) and ventral horns (IBA-1 g = −1.12 [95.0%CI −2.51, 0.578], *p* = 0.0976 and P2RY12 g = −0.906 [95.0%CI −2.92, 0.475], *p* = 0.158) (Figure 7A,B). Tregs also reversed the nerve-injury-induced inflammatory phenotypic shift, reducing LYZ expression (g = −0.949 [95.0%CI −2.16, 0.522], *p* = 0.106 Figure 7C) and increasing CD206 expression (g = 1.32 [95.0%CI 0.398, 2.18], *p* = 0.0142 Figure 8A) relative to IBA-1 and P2RY12, respectively. Tregs also reduced pain-resolving CD11c+IGF1+ cell density following both sham (g = −1.49 [95.0%CI −2.62, −0.233], *p* = 0.0244) and nerve injury (g = −2.16 [95.0%CI −3.18, −1.21], *p* = 0.0006 Figure 8B). However, following spinal delivery of Tregs, an increase in CD163+ cell density was seen in nerve-injured male mice relative to vehicle control mice in the ipsilateral dorsal horn (g = 0.861 [95.0%CI −0.584, 2.07], *p* = 0.194 Figure 8C).

In female mice, nerve injury resulted in an increase in microglia activation compared to sham-injured mice in ipsilateral dorsal (IBA-1 g = 1.36 [95.0%CI −0.404, 3.11], *p* = 0.0618 Figure 7D and P2RY12 g = 1.17 [95.0%CI −2.17, 5.41], *p* = 0.129 Figure 7E) and ventral horns (IBA-1 g = 1.12 [95.0%CI −1.49, 2.21], *p* = 0.109 and P2RY12 g = 1.1 [95.0%CI −0.535, 2.4], *p* = 0.143), with no change to LYZ expression relative to IBA-1 (Figure 7F). A reduction in CD206 expression (g = −1.83 [95.0%CI −3.68, −0.636], *p* = 0.0136 Figure 8D) and an increase in pain-resolving CD11c+IGF1+ cell density (dorsal g = 2.8 [95.0%CI 1.53, 3.82], *p* = 0.0136 and ventral g = 1.08 [95.0%CI 0.401, 1.88], *p* = 0.109 Figure 8E) was seen after CCI. Similar to what was observed in male mice, there was also a low count of CD11c+IGF1+ cells in the female spinal cord. Despite similar observations in microglial reactivity following CCI in male and female mice, Treg treatment did not alter microglia reactivity in female mice (Figure 7D,E). Similarly, there were no changes in CD206 relative expression (Figure 8D) or density of pain-resolving microglia (Figure 8E) following intrathecal injection of Tregs in nerve-injured female mice. Although CD163 cell density in the spinal cord parenchyma was low, Tregs reduced CD163 density in the spinal dorsal horn of sham mice (g = −3.58, [95.0%CI −4.37, −2.8], *p* < 0.0001) and no differences were found after CCI (Figure 8F).

### 3.7. Treatment with Tregs Reduces Neurotoxic Astrocytes in Males and Increases Neuroprotective Astrocytes in Female Mice

**Figure 9:**
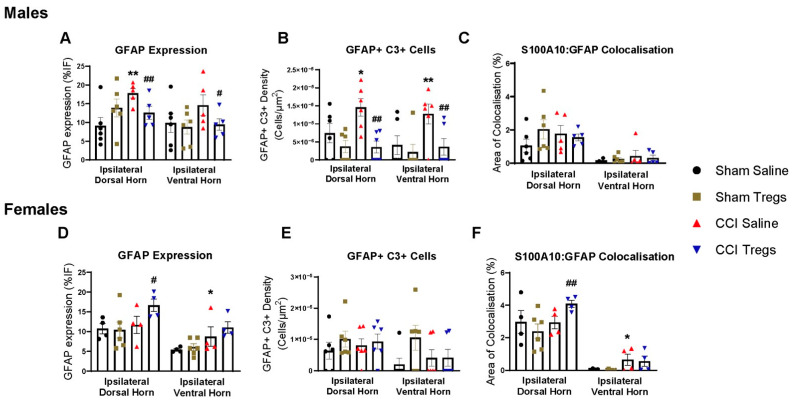
Changes in astrocyte phenotype in the ipsilateral spinal cord following intrathecal administration of Tregs in male and female mice. Nerve-injured and sham-operated mice were injected with activated Tregs or saline-based vehicles (control). Opal multiplex immunohistochemistry and immunofluorescent staining were used to assess astrocyte markers (**A**) GFAP, (**B**) C3+ GFAP+ cell density and (**C**) S100A10/GFAP colocalisation in males, and (**D**) GFAP, (**E**) C3+ GFAP+ cell density and (**F**) S100A10/GFAP colocalisation in females in the ipsilateral dorsal and ventral horns. Data are shown as mean ± SEM (* 0.8 < g < 1.2 and ** g > 1.2 for CCI relative to sham saline and # 0.8 < g < 1.2 and ## g > 1.2 relative to CCI saline, *n* = 4–6 per group).

To evaluate astrocytic responses, lumbar spinal sections were immunohistochemically stained for an astrocyte reactivity marker (GFAP), as well as markers for neurotoxic (C3) and neuroprotective (S100A10) astrocyte phenotypes (Figure 9). Representative images are illustrated in Figure S4, Figure S5, Figure S8 and Figure S9.

In males, nerve-injured mice exhibited an increase in astrocyte reactivity and neurotoxic astrocyte density relative to sham-injured mice in the ipsilateral dorsal horn (GFAP g = 1.77 [95.0%CI 0.241, 5.09], *p* = 0.0134 Figure 9A and GFAP+C3+ g = 1.06 [95.0%CI −0.231, 2.3], *p* = 0.0696 Figure 9B). Following Treg treatment in nerve-injured male mice, astrocyte reactivity and neurotoxic astrocytes in the ipsilateral dorsal horn decreased relative to vehicle-treated mice (GFAP g = −1.37 [95.0%CI −3.35, 0.164], *p* = 0.047 and GFAP+C3+ g = −2.01 [95.0%CI −3.38, −0.845], *p* = 0.0078). In the ipsilateral ventral horn, there was also an increase in neurotoxic astrocytes following nerve injury (GFAP+C3+ g = 1.21 [95.0%CI −0.175, 3.08], *p* = 0.0264), whilst Treg administration in CCI mice resulted in decreased astrocyte reactivity and density of neurotoxic astrocytes relative to vehicle-treated mice (GFAP g = −0.916 [95.0%CI −2.06, 0.406], *p* = 0.168 and GFAP+C3+ g = −1.35 [95.0%CI −3.32, 0.173], *p* = 0.0116). There was no change in neuroprotective astrocytes in male mice (Figure 9C).

In females, nerve injury did not alter GFAP expression in the ipsilateral dorsal horn; however, Treg treatment in CCI mice resulted in increased astrocyte reactivity (GFAP g = 1.14 [95.0%CI −0.236, 2.52], *p* = 0.125 Figure 9D) and neuroprotective astrocyte expression (GFAP/S100A10 colocalisation g = 1.58 [95.0%CI 0.233, 2.91], *p* = 0.0528 Figure 9F) relative to CCI vehicle-treated mice. In the ipsilateral ventral horn, nerve injury increased astrocyte reactivity (GFAP g = 0.824 [95.0%CI −1.07, 1.36], *p* = 0.0662 Figure 9D) and neuroprotective astrocytes (GFAP/S100A10 colocalisation g = 1.08 [95.0%CI −0.326, 14.4], *p* = 0.15 Figure 9F) in CCI vehicle-treated mice relative to sham-injured female mice. There was no change in neurotoxic astrocytes in female mice (Figure 9E).

## 4. Discussion

It is now well-recognised that males and females differ in their innate and adaptive immune responses that relate to the pain experience [39]. Several preclinical studies across males and females have demonstrated a beneficial role for Tregs in the resolution of chronic pain [9], with a recent study reporting sexual dimorphism in the interactions of Tregs and microglia that regulate pain hypersensitivity [27]. Here, comparing the effects of intrathecal adoptive transfer of Tregs in male and female mice following CCI, we investigated Treg-induced changes in immune cell profiles in the meninges, choroid plexus, and peripheral lymphatic tissues, along with spinal glial phenotypes. Tracking the adoptively transferred Tregs, we observed that the injected cells accumulated predominantly in the choroid plexus, the pia mater, and the cervical, inguinal, and sciatic/popliteal LNs, suggesting they have drained from the CNS borders to the peripheral lymphatic system in both males and females. The injection of Tregs resulted in decreased pain behaviours in both sexes but in differential changes to immune cell profiles centrally and peripherally in males and females, such as reduced total leukocytes in the meninges in male mice and increased Tregs and effector memory T cells in female mice. Moreover, in the spinal cord, a sex-dependent response of glial cells was identified. Treg treatment reduced inflammatory microglial reactivity and neurotoxic astrocytes in male mice whilst increasing the expression of neuroprotective astrocytes in female mice.

Intrathecal administration of GFP+ Tregs led to an accumulation of these cells within the pia 48 h later. These GFP+ Tregs remain upregulated within the pia up to 6 days after delivery, though the total number of GFP+ Tregs was remarkably lower. Despite remaining in the pia, we did not observe an infiltration of GFP+ Tregs into the spinal cord parenchyma. Rather, GFP+ Tregs migrated into the choroid plexus and the draining cervical and inguinal LNs, indicating drainage from the meninges into the peripheral lymphatic system. The intrathecal route of administration reaches the CSF, and the total volume of the CSF is recycled several times per day [30]. Studies using fluorescent and radiolabeled tracer injections into the CSF demonstrated tracer influx from the subarachnoid compartment into the brain parenchyma along paravascular spaces, dependent on molecular weight [40]. Subsequent studies showed that 30 min after intracerebroventricular dye injection, the dye was already detected in the meningeal lymphatic vessels and drained into the CLN [31]. T cells also drain from the meningeal spaces to CLN through the meningeal lymphatic vessels, and resection of the deep CLN results in an accumulation of meningeal T cells [31]. In addition, an extensive network of vertebral epidural and dural lymphatic vessels drains the vertebral canal toward lymph nodes adjacent to the spinal cord, and these vertebral lymphatic vessels are associated with CD45+ leukocytes [32]. Consistent with such lymphatic vessel-mediated drainage of the vertebral column, we found increased GFP+ Tregs infiltrating the sciatic/popliteal LNs after CCI in both male and female mice. These data highlight that intrathecally delivered cells can reach the different meningeal compartments, traffic out by meningeal lymphatic vessels, and enter peripheral lymph nodes.

Our behavioural results demonstrated that peripheral nerve injury resulted in mechanical pain hypersensitivity in male and female mice, with an apparent lower paw withdrawal threshold in females. Nerve injury-induced changes in exploratory behaviours were also observed in males and females, with more pronounced reduced nose poking in males and increased anxiety-like behaviour (reduced time spent in the centre area of the open field) in females. It is noteworthy that sham-operated mice also showed some degree of pain hypersensitivity during the experimental period, as the surgery is expected to induce postsurgical pain and avoidance behaviour [41]. Importantly, our findings confirm previous studies showing that Treg administration is sufficient to alleviate neuropathic pain-induced behavioural changes in both male and female mice following peripheral nerve injury [14,15,16,18]. However, despite similar effects on pain behaviours, Treg adoptive transfer had a differential impact on peripheral and meningeal immunity in males and females.

Interestingly, peripheral nerve injury also induced sex-dependent immune changes in the meninges and lymph nodes, consistent with sex differences in the activity of innate and adaptive immune responses [42]. In the peripheral lymphatic system, we found increases in total leukocytes, T helper cells, cytotoxic T cells, B cells and DCs in sciatic/popliteal LNs in male mice following CCI. Additionally, nerve injury expanded FoxP3+ Tregs only in female mice centrally (pia mater) and peripherally (LNs and spleen). Spinal delivery of Tregs led to the accumulation of GFP+ Tregs in the sciatic/popliteal LNs in both sexes of nerve-injured mice and in the inguinal and cervical LNs of female mice, as well as noticeably suppressing cytotoxic T cells in male mice. Previous studies have demonstrated that peripheral nerve injury modulates the immune response in LNs. For example, sciatic denervation in mice causes loss of sympathetic innervation to the popliteal LN, inducing LN expansion due to interferon-γ expression in CD8+ T cells [43]. Ablation of sympathetic innervation in the periphery increases splenic and LN Tregs in EAE and lymphoproliferative disease mouse models [44,45]. The infiltration or expansion of Tregs at the site of peripheral nerve injury alleviates neuropathic pain [14,16]. Specifically, following partial nerve ligation of the sciatic nerve, Tregs accumulate at the injury site and secrete IL-10, suppressing CD4+ Th1 cells and their effector cytokine interferon-ɣ, alleviating mechanical pain [16]. Although we did not investigate CD4+ T helper cell subpopulations, we cannot determine whether the infiltrating GFP+ Tregs specifically suppressed proinflammatory Th1 cells. Tregs are known as potent suppressors of effector T cells [10]. Indeed, expanding Tregs via CD28 superagonist treatment decreases the numbers of infiltrating T cells, macrophages and antigen-presenting cells in the sciatic nerve and dorsal root ganglia [14]. Therefore, it is likely that the Tregs migrating from the draining LNs of the sciatic nerve (sciatic/popliteal LNs) to the injured nerve are suppressing neuroinflammatory responses and consequently alleviating neuropathic pain.

In recent years, the meninges have emerged as fundamental immunological niches with T-cell activation by local antigen-presenting cells and cytokine release by various immune cells, which affect CNS function [28,30]. In addition, distinct roles of meningeal layers have been shown in CNS autoimmunity [37]. Our findings of immune cell populations in the CNS borders also showed different meningeal layer and choroid plexus tissue involvement. We found increases in total leukocytes and several immune cell populations in the dura/arachnoid mater and pia mater in EAE-affected animals compared to control mice, consistent with general EAE pathogenesis. Indeed, meningeal inflammation precedes the onset of clinical symptoms in EAE [46]. Tertiary lymphoid clusters of T cells are found in meningeal tissues of patients with multiple sclerosis and mice with EAE, correlating with disease severity [47]. Treg administration into EAE-affected mice resulted in reduced effector T cells and DCs in the dura/arachnoid mater, pia mater and choroid plexus. These findings suggest a shift towards an anti-inflammatory environment associated with reduced neuroinflammation and pain behaviours in Treg-treated mice with EAE [12]. While intrathecal delivery of activated Tregs in mice with EAE resulted in increased GFP+ Tregs in the pia mater, there was an unexpected reduction in FoxP3+ cells in this tissue, which may be attributed to non-specific FoxP3+ expression in EAE. FoxP3 is transiently expressed by non-regulatory T cells following activation [48], as well as B cells, natural killer cells, and macrophages [49].

Following CCI, we observed an expansion of leukocytes in the meninges (pia and dura mater) of male mice. Nerve injury is reported to induce the infiltration of leukocytes into the lumbar dorsal root leptomeninges of male rodents, specifically CD4+ T cells [50] in rats following tibial nerve injury and CD11c+ dendritic cells [51] in mice after spared nerve injury. Partial sciatic nerve ligation has previously been reported to disrupt the blood-spinal cord barrier in rats, increasing permeability and influx of inflammatory mediators [52]. Interestingly, the influx of CD4+ T cells and dendritic cells into the leptomeninges of male rodents contributes to the development of neuropathic pain via activation of spinal glial cells [50,51], highlighting the capacity for meningeal or perivascular cells to regulate glial cell reactivity without infiltrating the parenchyma [28,30]. Surprisingly, in the choroid plexus, we observed a reduction in FoxP3+ Tregs, monocytes/macrophages and CD11b+ DCs. This contrasts a previous study that reported increased choroid plexus CD68+ and CD163+ macrophages in rats following CCI [53]. The meningeal immune profile differed in female mice, displaying reduced monocytes/macrophages, an increase in FoxP3+ Tregs in the pia, and an increase in cytotoxic T cells in the choroid plexus following nerve injury. The increased Tregs in the pia mater of nerve-injured female mice is consistent with a previous report demonstrating a preferential expansion of Tregs in spinal cord meninges in females after intrathecal injection of CSF-1 [27], indicating that female mice may have a higher capacity for expansion of endogenous meningeal Tregs post-injury. Administration of Tregs resulted in accumulating GFP+ Tregs in the choroid plexus and pia in male mice, suppressing the nerve injury-induced upregulation of the leukocyte population in the meninges. In nerve-injured female mice, GFP+ Tregs were found in the choroid plexus, and Treg treatment resulted in an increase in FoxP3+ Tregs and effector memory T cells in the dura/arachnoid tissue. Our findings highlight sex differences in immune cell profiles in the meningeal tissues and choroid plexus after peripheral nerve injury.

Spinal microgliosis, a feature of peripheral nerve injury [54], differs in its effects between male and female mice. Microglial activation in the spinal cord is critical for the development of neuropathic pain in male but not female mice [26,55], and transcriptional profiling of spinal microglia shows more robust phagosome, lysosome, and inflammatory activation in the acute phase after injury in males [56,57]. In both male and female mice, we observed increased spinal microglial reactivity ten days after CCI. However, only male mice showed an inflammatory phenotypic shift, characterised by increased expression of LYZ (a lysosome marker). In contrast, both male and female mice had reduced expression of the M2-like marker CD206. CD206, known as a mannose receptor, is a 175-kDa transmembrane protein broadly recognised as a typical M2 microglial marker and is induced following minocycline treatment in nerve injury models, indicating a shift from inflammatory to anti-inflammatory microglial activation [58,59]. We also observed an increase in the recently described CD11c+IGF1+ pain-resolving microglia in male and female nerve-injured mice [60]. CD11c+IGF1+ microglia appear at two weeks and peak at five weeks after peripheral nerve injury, and their depletion hinders spontaneous recovery during the chronic pain phase (day 14 onwards) [60]. Although we observed changes in their density, the total number of cells identified at the acute stage was extremely low, suggesting a limited biological impact.

Following spinal delivery of Tregs, nerve-injured male mice show a reduction in microglial reactivity and inflammatory activation (reduced LYZ expression) and increased CD206 expression relative to untreated mice, indicative of a phenotypic shift from inflammatory M1-like to anti-inflammatory M2-like microglia [58,59]. In contrast, female mice exhibited no changes in microglial reactivity following Treg administration, aligning with our previous study demonstrating that adoptive transfer of Tregs following EAE did not alter IBA-1 expression in female mice [12]. Evidence shows that Tregs suppress microglial reactivity and inflammatory activation through the secretion of their effector cytokines, IL-10 and TGFβ [61,62]. Specifically, spinal IL-10 administration alleviates mechanical pain. It reduces IBA-1 expression in the ipsilateral dorsal horn [63,64], and upregulating release of IL-10 from CD163+ spinal macrophages attenuates spinal microglial reactivity and mechanical pain in male mice after nerve injury [38]. Intrathecal administration of TGFβ also attenuates thermal hyperalgesia and reduces spinal microglial reactivity and inflammation after CCI [65]. Therefore, Tregs in the meninges of male mice following CCI and Treg administration may induce an anti-inflammatory shift in microglial phenotype via Treg effector cytokines IL-10 and TGFβ. 

Our finding of a robust sex-dependent microglial response to Treg administration following nerve injury is consistent with a previous study demonstrating that microglial activation was induced selectively in male mice after intrathecal injection with CSF-1 (in the absence of injury) [27]. CSF-1 selectively targets microglia, which express the receptor CSF-1R, in the spinal cord parenchyma [27]. Further, knocking out *Csf1* in female mice did not alter the development of mechanical pain following peripheral nerve injury [27,66]. In the spinal cord leptomeninges, female mice demonstrated a larger expansion of Tregs compared to males. Depleting Tregs in female mice resulted in increased mechanical pain and induced microglial activation, resembling the observations seen in male mice following intrathecal CSF-1 injection [27]. Similarly, in our study, female mice displayed a more robust increase in FoxP3+ Tregs in the pia after CCI than males. CSF-1R regulates interferon-related transcription factors interferon regulatory factor (IRF)-5 and IRF8 [66]. These interferon-related transcription factors induce microglial activation via upregulation of P2XR4 in microglia [67], which is critical for the development of mechanical pain hypersensitivity in males but not in female rodents [26,68,69]. Therefore, the sex-dependent effect of the adoptive transfer of Tregs may be related to a limited endogenous meningeal Treg expansion in males and the suppression of microglial activation via the CSF-1R-IRF5/8-P2XR4 axis.

The presence of anti-inflammatory (CD206+CD163+) meningeal macrophages is implicated in pain resolution [9]. Whilst tissue injury promotes their expansion, peripheral nerve injury blunts this upregulation of meningeal CD206+CD163+ cells [38]. Further, the upregulation of CD163 in spinal macrophages enhanced the production of IL-10, attenuated spinal microglial and astrocyte reactivity, and alleviated mechanical pain in male mice after nerve injury [38]. Our results show that administration of Tregs increased CD163+ cells in the spinal cord dorsal horn in male mice, with no changes in CD163+ cells observed in female mice. A decrease in microglia and astrocyte reactivity accompanied this increase in CD163+ cells in male mice. Since spinal delivery of Tregs increases spinal IL-10 expression [12] and co-culturing macrophages with Tregs in vitro upregulates CD163 expression in human macrophages [70], spinal delivery of Tregs may promote the resolution of neuropathic pain via IL-10-induced upregulation of CD163+ macrophages, suppressing microglial and astrocyte reactivity in males.

Reactive astrocytes are implicated in the maintenance of neuropathic pain following peripheral nerve injury [71], with evidence suggesting they play a critical role in both male and female mice [72]. Further, spinal delivery of Tregs attenuated GFAP expression in the trigeminal nuclei of EAE female mice [12]. Our results show that astrocyte reactivity is more prominent in male mice, with GFAP expression increasing in the dorsal and ventral horn ten days after CCI. Further, only male mice displayed increased neurotoxic astrocytes (GFAP+C3+) following CCI. Neurotoxic astrocytes are induced by inflammatory microglia [73] and can secrete neurotoxins that impair neuronal and oligodendrocyte function. Neurotoxic astrocytes are upregulated in the spinal cord in a model of chronic post-surgical pain [74], where treatment with the microglial-inhibitor minocycline attenuated the development of mechanical pain and the expression of neurotoxic astrocytes (C3+) and increased the expression of neuroprotective astrocytes (S100A10+) [74].

Interestingly, in male mice, we found that Treg treatment attenuated astrocyte reactivity (GFAP expression) and reduced neurotoxic astrocytes but did not alter the levels of neuroprotective S100A10+/GFAP+ astrocytes. Contrastingly, in female mice, Treg treatment increased astrocyte reactivity (GFAP expression) and neuroprotective S100A10+/GFAP+ astrocytes. Tregs have previously been shown to suppress neurotoxic astrogliosis in the mouse brain, potentiating neurological recovery after an ischaemic stroke [75]. Treg-secreted IL-10 could also contribute to reduced astrocyte reactivity, as IL-10 treatment reduces spinal astrocyte reactivity in a chronic inflammatory pain model [63]. While the finding of Treg-induced sex-dependent divergent astrocyte response following CCI is unexpected, astrocytes exhibit distinct reactive changes in males and females in response to pathological insults, both in vitro [76,77] and in vivo [78,79]. Previous studies suggest that steroid hormones play a role. In cerebral ischemia, neuron- or astrocyte-derived 17β-estradiol regulates neuroprotective astrocyte activation and upregulates S100A10 following brain injury [80,81]. Furthermore, 17β-estradiol administration following CCI reduced mechanical pain and improved functional recovery in female mice [82]. These improved outcomes following treatment were accompanied by an increase in GFAP expression in the spinal cord, which was not observed in male mice [82]. These findings highlight that reactive astrocytes have a bilateral role in neuropathic pain, and different subtypes of reactive astrocytes might play sex-dependent roles in Treg-mediated pain resolution.

## 5. Conclusions

Overall, a deeper understanding of the effects of Tregs on nerve injury-induced immune responses in males and females is essential for the development of Treg therapeutic strategies in neuropathic pain. Here, we demonstrate sex-specific changes in meningeal and peripheral LN immunity and spinal glial reactivity following CCI in mice. We also show that following intrathecal delivery, Tregs initially accumulate in the pia mater before dispersing into the choroid plexus and peripheral LNs 6 days after injection. Treg treatment alleviates pain behaviours in both males and females after CCI, although it results in differential changes in the meningeal and peripheral LN immunity. Despite not entering the spinal cord parenchyma, Tregs also alter spinal glial phenotypes, suppressing inflammatory microglia and neurotoxic astrocytes in males and promoting neuroprotective astrocytes in females. Therefore, we suggest that the adoptive transfer of Tregs facilitates the resolution of neuropathic pain via different mechanisms in males and females. Cellular therapies utilising Tregs have been shown to have therapeutic potential in various inflammatory and autoimmune diseases and transplant rejection [83,84]. Given the accumulating evidence that Tregs promote pain resolution in preclinical neuropathic pain models, Treg therapy is a compelling therapeutic solution for treating some neuropathic pain syndromes. However, further research is required to elucidate the sex-specific mechanisms by which Tregs regulate neuropathic pain to enable their development as potential tools for pain management.

## Data Availability

The data presented in this study are available on request from the corresponding author.

## Supplementary Materials

The following supporting information can be downloaded at: https://www.mdpi.com/article/10.3390/cells12182317/s1. Supporting information Figures S1–S9 is enclosed.
