# Supplementary material for: Differential Effects of Regulatory T Cells in the Meninges and Spinal Cord of Male and Female Mice with Neuropathic Pain
Source: Cells. 2023 Sep 20;12(18):2317. doi: 10.3390/cells12182317 (PMC10527659; doi:10.3390/cells12182317)
Supplement: Supplementary file 1 [file cells-12-02317-s001.zip › cells-2491879-supplementary.pdf]

SUPPLEMENTARY MATERIAL - 'Differential Effects of Regulatory T Cells in the Meninges and Spinal Cord of Male and Female Mice with Neuropathic Pain'

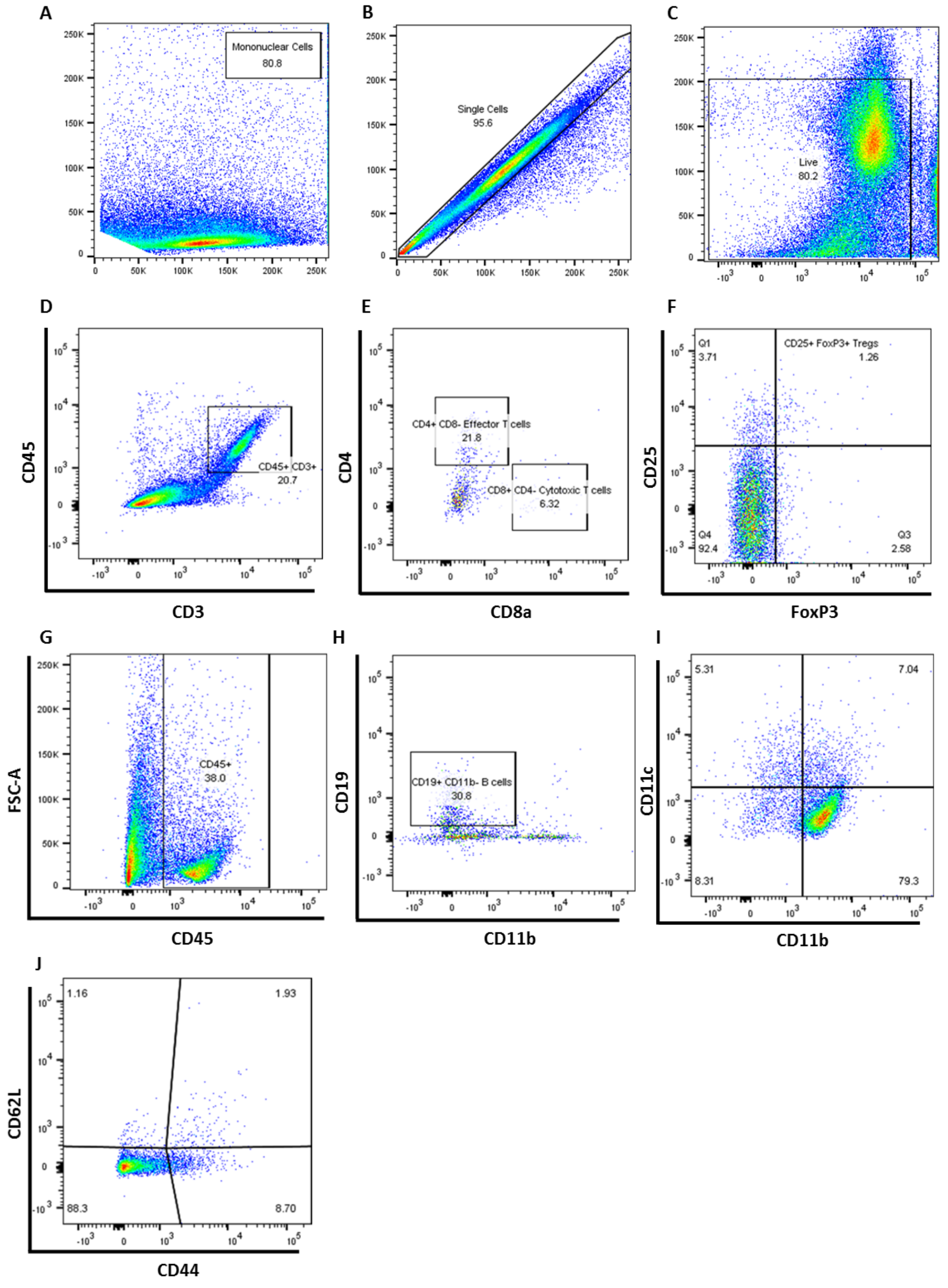

Figure S1. Gating strategy used for flow cytometric analysis. (A) Mononuclear cells were first gated, followed by singlets (B) and live cells (C). All subsequent gates stem from the "live cell" parent gate. For effector T cells, CD45 and CD3 double positive cells were gated

(D), followed by (E) CD4+ CD8a- and CD8a+ CD4- gating for T helper cells and cytotoxic T cells, respectively. For Tregs (F), CD25 and FoxP3 double positive cells were gated from the "T helper cell/effector T cell" parent gate. For B cells and myeloid cells, CD45+ cells alone were gated from the "live cell" parent gate (G), followed by CD19+ CD11b- gating for B cells (H). CD11c and CD11b were used for DCs and monocytes/macrophages (I), with CD11c+ CD11b- (top left quadrant) used for CD11b- DCs, CD11c+ CD11b+ (top right quadrant) used for CD11b+ DCs, and CD11b+ CD11c- (bottom right quadrant) used for monocytes/macrophages. (J) Memory T cells were either gated from their CD4+ or CD8a+ parent gate for CD62L lo CD44 hi expression (bottom right quadrant) for Tem cells, or CD62Lhi CD44 hi expression (top right quadrant) for Tcm cells.

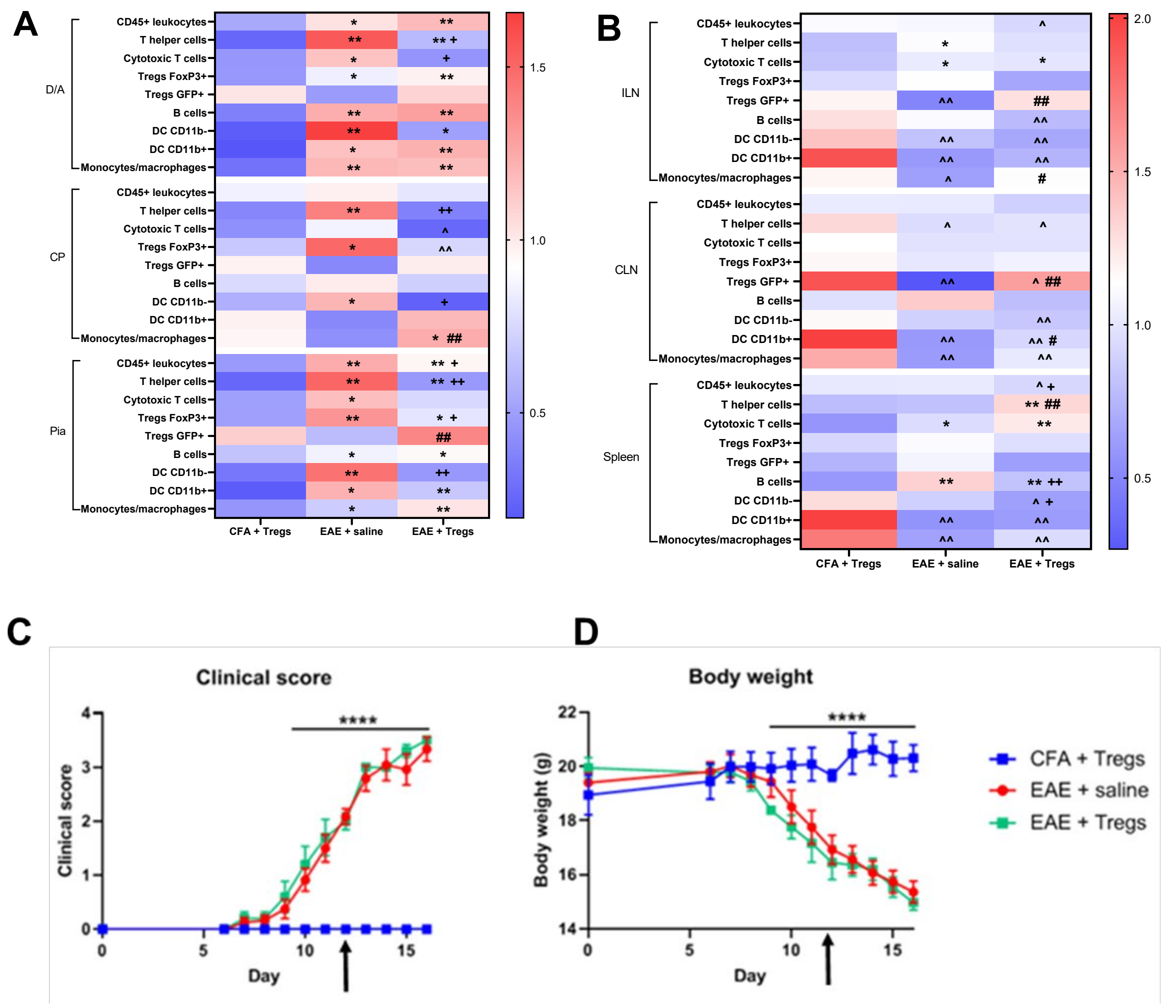

Figure S2. Intrathecal administration of activated Tregs modulates immune cell profile in the meninges, choroid plexus, and lymphoid organs in EAE, but does not affect disease progression when delivered following symptom onset.

Heatmap summarising immunological changes in the meninges and choroid plexus **(A)** and peripheral lymphatic tissue **(B)** in EAE 4 days following intrathecal delivery of activated Tregs or saline-based vehicle (control). Data normalised for heatmaps. Raw data analysed using estimation statistics (* 0.8 < g < 1.2 and ** g > 1.2 for Treg-recipient mice compared to the saline vehicle; # for EAE + Tregs mice compared to EAE + saline; ^ for EAE-affected mice compared to CFA mice; + for EAE + Tregs mice compared to EAE + saline mice; *n* = 5-6). D/A = dura/arachnoid, CP = choroid plexus.

(A) In the dura/arachnoid, increases were seen in both EAE groups compared to the control CFA + Tregs group in the proportion of CD45+ leukocytes (EAE + saline: g = 0.879 [95.0%CI -0.0325, 1.66], p = 0.117; EAE + Tregs: g = 2.03 [95.0%CI 0.675, 3.39, p = 0.008), T helper cells (EAE + saline: g = 1.41 [95.0%CI 0.668, 2.15], p = 0.0156; EAE + Tregs: g = 2.16 [95.0%CI 0.802, 3.79], p = 0.0158), FoxP3+ Tregs (EAE + saline: g = 1.1 [95.0% CI 0.169, 1.98], p = 0.0596; EAE + Tregs: g = 1.22 [95.0%CI -0.323, 2.77], p = 0.057), B cells (EAE + saline: g = 1.25 [95.0%CI 0.0792, 2.37], p = 0.0418; EAE + Tregs: g = 1.52 [95.0%CI 0.111, 3.07], p = 0.0388), CD11b- DCs (EAE + saline: g = 1.29 [95.0%CI 0.621, 2.25], p = 0.0302; EAE + Tregs: g = 1.1 [95.0%CI -0.104, 1.77], p = 0.0424), CD11b+ DCs (EAE + saline: g = 1.2 [95.0%CI 0.0641, 2.12], p = 0.0462; EAE + Tregs: g = 1.79

[95.0%CI 0.0977, 4.49], p = 0.0158), and monocytes/macrophages (EAE + saline: g = 1.26 [95.0%CI 0.4, 2.05], p = 0.0272; EAE + Tregs: g = 2.26 [95.0%CI 1.07, 3.63], p < 0.0001). Cytotoxic T cells were increased in the dura/arachnoid in EAE + saline animals compared to CFA + Tregs (g = 0.881 [95.0%CI 0.18, 1.4], p = 0.119). Decreases in the dura/arachnoid were seen in both T helper cell (g = -1.02 [95.0%CI -1.78, -0.199], p = 0.0804) and cytotoxic T cell populations (g = -0.883 [95.0%CI -1.4, -0.252], p = 0.123) in EAE + Tregs mice compared to EAE + saline animals. In the choroid plexus, T helper cells (g = 1.41 [95.0%CI 0.68, 2.35], p = 0.0164), FoxP3+ Tregs (g = 1.13 [95.0%CI 0.0809, 2.23], p = 0.0512), and CD11b- DCs (g = 0.954 [95.0%CI 0.387, 1.52], p = 0.0696) were increased in EAE + saline mice compared to CFA + Tregs control animals. Monocytes/macrophages were also increased in EAE + Tregs animals compared to CFA + Tregs mice (g = 0.839 [95.0%CI -0.573, 1.94], p = 0.149), as well as compared to EAE + saline animals (g = 1.32 [95.0%CI 0.0424, 2.67], p = 0.0294). Cytotoxic T cells (g = -1.03 [95.0%CI -2.8, 0.395], p = 0.0936) and FoxP3+ Tregs (g = -1.47 [95.0%CI -2.58, -0.441], p = 0.0192) were decreased in the choroid plexus in EAE + Tregs mice compared to CFA + Tregs control mice, and T helper cells (g = -1.46 [95.0%CI -2.37, -0.76], p = 0.0114) and CD11b- DCs (g = -1.12 [95.0%CI -1.71, -0.484], p = 0.0432) were also reduced in EAE + Tregs mice compared to EAE + saline animals. In the pia, increases in both EAE groups compared to CFA + Tregs animals were seen in CD45+ leukocytes (EAE + saline: g = 2.15 [95.0%CI 1.17, 3.1], p = 0.0014; EAE + Tregs: g = 3.43 [95.0%CI 1.76, 6.88], p < 0.0001), T helper cells (EAE + saline: g = 1.82 [95.0%CI 1.07, 2.63], p = 0.0034; EAE + Tregs: g = 1.38 [95.0%CI -0.239, 3.29], p = 0.0496), FoxP3+ Tregs (EAE + saline: g = 1.86 [95.0%CI 0.863, 2.92], p = 0.0044; EAE + Tregs: g = 0.973 [95.0%CI -0.3, 2.3], p = 0.15), B cells (EAE + saline: g = 0.823 [95.0%CI 0.245, 1.33], p = 0.129; EAE + Tregs: g = 1.09 [95.0%CI -0.334, 1.85], p = 0.0606), CD11b+ DCs (EAE + saline: g = 1.17 [95.0%CI 0.64, 1.65], p = 0.0288; EAE + Tregs: g = 1.26 [95.0%CI 0.211, 2.18], p = 0.0382), and monocytes/macrophages (EAE + saline: g = 0.823 [95.0%CI 0.322, 1.41], p = 0.118; EAE + Tregs: g = 3.55 [95.0%CI 2.96, 4.05], p = 0.006). Increases were seen in cytotoxic T cells (g = 0.954 [95.0%CI 0.454, 1.64], p = 0.059) and CD11b- DCs (g = 1.58 [95.0%CI 0.719, 2.53], p = 0.0084) in EAE + saline mice compared to CFA animals, while GFP+ Tregs were increased in EAE + Tregs mice compared to EAE + saline (g = 1.57 [95.0%CI -0.315, 2.82], p = 0.0042). Several immune cell populations were also decreased in the pia of EAE + Tregs mice when compared to EAE + saline animals, including the proportion of CD45+ leukocytes (g = -0.804 [95.0%CI -1.56, 0.0924], p = 0.145), T helper cells (g = -1.48 [95.0%CI -2.27, -0.774], p = 0.0132), FoxP3+ Tregs (g = -1.18 [95.0%CI -2.13, -0.267], p = 0.0394), and CD11b- DCs (g = -1.33 [95.0%CI -2.23, -0.457], p = 0.0204).

(B) In the inguinal LNs, T helper cells were increased in EAE + saline mice (g = 0.876 [95.0%CI 0.0881, 1.79], p = 0.108), while cytotoxic T cells were increased in both EAE groups (EAE + saline: g = 0.946 [95.0%CI 0.00974, 2.03), p = 0.0862; EAE + Tregs: g = 0.957 [95.0%CI -0.432, 2.13], p = 0.138), compared to CFA + Tregs animals. GFP+ Tregs were also elevated in EAE + Tregs animals compared to EAE + saline mice (g = 1.32 [95.0%CI -0.0559, 2.79], p = 0.0132), as well as monocytes/macrophages (g = 1.02 [95.0%CI -0.209, 2.41], p = 0.0598). Decreases in the proportion of CD45+ leukocytes (g = -1.03 [95.0%CI -1.88, 1.03], p = 0.0856) and B cells (g = -1.48 [95.0%CI -2.94, 0.101], p = 0.0288) were also observed in the inguinal LNs of EAE + Tregs mice, while GFP+ Tregs (g = -1.42 [95.0%CI -3.2, -0.107], p = 0.0124) and monocytes/macrophages (g = -1.06 [95.0%CI -2.92, 0.124], p = 0.0558) were simultaneously decreased in EAE + saline animals, compared to CFA + Tregs mice. CD11b- (EAE + saline: g = -1.69 [95.0%CI -2.66, -0.783], p = 0.0054; EAE + Tregs: g = -2.94 [95.0%CI -3.8, -2.19], p < 0.0001) and CD11b+ DCs (EAE + saline: g = -5.41 [95.0%CI -8.66, -3.53], p = 0.0002; EAE + Tregs: g = -3.49 [95.0%CI -4.98, -2.2],

p = 0.0096) were also decreased in both EAE groups compared to CFA + Tregs animals. In the cervical LNs, increases were only seen in GFP+ Tregs (g = 1.85 [95.0%CI -0.799, 5.34], p = 0.0092) and CD11b+ DCs (g = 1.07 [95.0%CI -1.05, 2.72], p = 0.0692) in EAE + Tregs mice compared to EAE + saline animals; however, decreases were seen in several cell populations. Compared to CFA + Tregs mice, decreases were seen in both EAE groups in T helper cells (EAE + saline: g = -0.994 [95.0%CI -1.88, 0.271], p = 0.0806; EAE + Tregs: g = -0.922 [95.0%CI -2.14, 0.713], p = 0.159), GFP+ Tregs (EAE + saline: g = -4.68 [95.0%CI -7.98, -2.8], p = 0.0002; EAE + Tregs: g = -0.912 [95.0%CI -1.81, 0.606], p = 0.0852), CD11b+ DCs (EAE + saline: g = -6.55 [95.0%CI -8.1, -5.08], p = 0.0002; EAE + Tregs: g = -3.63 [95.%CI -4.71, -2.59], p < 0.0001), and monocytes/macrophages (EAE + saline: g = -2.0 [95.0%CI -3.93, -0.578], p = 0.0032; EAE + Tregs: g = -1.74 [95.0%CI -2.97, -0.601], p = 0.0268), while CD11b- DCs were reduced in EAE + Tregs mice compared to CFA + Tregs mice (g = -1.71 [95.0%CI -2.39, -1.02], p < 0.0001). In the spleen, T helper cells were increased in EAE + Tregs mice compared to both CFA + Tregs (g = 2.45 [95.0%CI 0.87, 6.33], p = 0.0186) and EAE + saline groups (g = 1.35 [95.0%CI 0.249, 3.01], p = 0.0262), while in both EAE groups compared to CFA + Tregs mice increases in cytotoxic T cells (EAE + saline: g = 1.19 [95.0%CI 0.592, 1.81], p = 0.0494; EAE + Tregs: g = 4.57 [95.0%CI 2.98, 6.38], p = 0.0002) and B cells (EAE + saline: g = 2.06 [95.0%CI 1.02, 3.95], p = 0.0044; EAE + Tregs: g = 2.17 [95.0%CI 0.916, 3.4], p = 0.0068) were seen. Conversely, decreases were seen in CD45+ leukocytes (EAE + saline: g = -1.16 [95.0%CI -2.63, 0.155], p = 0.0416; CFA + Tregs: g = -0.815 [95.0%CI -2.25, 1.31], p = 0.219) and CD11b- DCs (EAE + saline: g = -0.821 [95.0%CI -1.45, -0.248], p = 0.135; CFA + Tregs: g = -2.8 [95.0%CI -6.43, -1.03], p = 0.0022) in EAE + Tregs mice compared to both EAE + saline and CFA + Tregs mice, and B cells were also decreased in EAE + Tregs mice compared to EAE + saline animals (g = -1.32 [95.0%CI -2.74, -0.283], p = 0.0268). CD11b+ DCs (EAE + saline: g = -2.8 [95.0%CI -4.62, -1.54], p < 0.0001; EAE + Tregs: g = -3.72 [95.0%CI -5.26, -2.43], p = 0.003) and monocytes/macrophages (EAE + saline: g = -3.03 [95.0%CI -5.53, -1.47], p = 0.0004; EAE + Tregs: g = -2.75 [95.0%CI -5.22, -1.25], p = 0.012) were reduced in both EAE groups compared to CFA + Tregs control animals.

EAE clinical score **(C)** and body weight **(D)** of female C57BL/6J mice over the 16-day monitoring period. Black arrows denote the time of intrathecal delivery of activated Treg or saline-based vehicle (i.e. day 12). ****P < 0.0001. Repeated-measures two-way ANOVA with Tukey's correction for multiple comparisons.

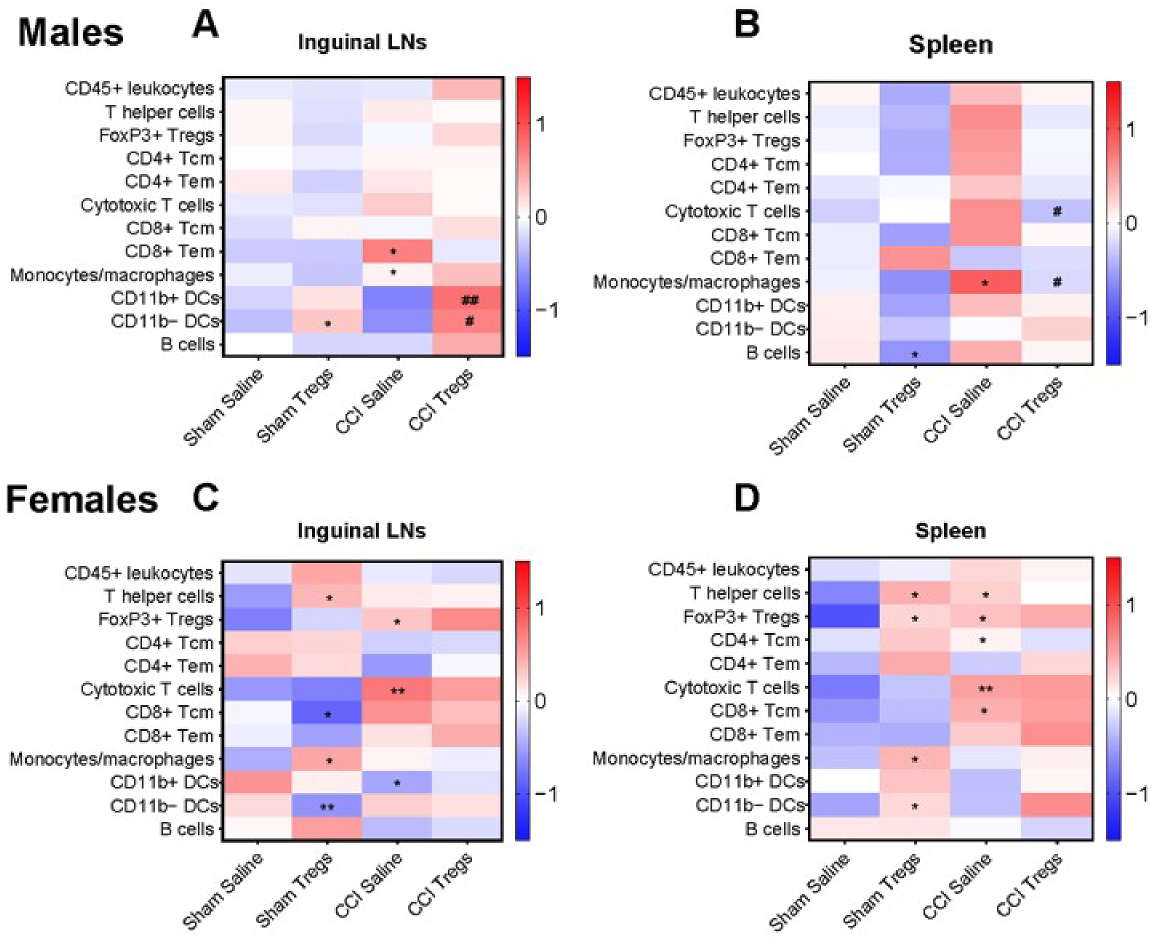

Figure S3. Immune cell profiles in the inguinal lymph node and spleen following peripheral nerve injury and intrathecal Treg injection in male and female mice.

Heatmaps summarising immunological changes in the **(A)** inguinal LNs and **(B)** spleen of male mice and **(C)** inguinal LNs and **(D)** spleen of female mice following CCI or sham operation and intrathecal administration of activated Tregs or saline-based vehicle (control). Data normalised for the heatmaps. Raw data analysed using estimation statistics. (* $0.8 < g < 1.2$ and ** $g > 1.2$ for differences relative to sham saline and # $0.8 < g < 1.2$ and ## $g > 1.2$ relative to CCI saline, n = 7-10).

In males, in the inguinal LNs **(A)**, CD11b- DCs were increased following Treg injection in sham mice (g = 0.934 [95.0%CI -0.459, 2.55], p = 0.114), and CD11b- DCs (g = 1.02 [95.0%CI -0.099, 2.27], p = 0.0854) and CD11b+ DCs (g = 1.34 [95.0%CI 0.441, 2.0], p = 0.0102) were increased in CCI mice injected with Tregs as compared to saline-treated animals. In the spleen **(B)**, compared to sham + saline animals, B cells were decreased in sham + Tregs mice (g = -0.814 [95.0%CI -1.95, 0.775], p = 0.159), while monocytes/macrophages were increased in CCI + saline mice (g = 0.827 [95.0%CI -0.558, 2.19], p = 0.145). As well, cytotoxic T cells (g = -0.852 [95.0%CI -2.27, 0.438], p = 0.15) and monocytes/macrophages (g = -1.17 [95.0%CI -3.07, -0.0042], p = 0.0618) were decreased in CCI + Tregs animals compared to CCI + saline.

In females, in the inguinal LNs **(C)**, nerve-injured mice displayed increases in cytotoxic T cells (g = 1.21 [95.0%CI -0.109, 2.87], p = 0.044), and FoxP3+ Tregs (g = 0.938 [95.0%CI -0.338, 3.31], p = 0.102) relative to sham mice, while CD11b+ DCs (g = -0.861 [95.0%CI -2.04, 0.502], p = 0.132) and CD4+ Tem cells were reduced (g = -1.02 [95.0%CI -2.34,

0.515], p = 0.0744). Following Treg injection in sham mice, an increase was seen in T helper cells (g = 0.939 [95.0%CI -0.192, 1.98], p = 0.0892) and monocytes/macrophages (g = 0.881 [95.0%CI -0.378, 1.74], p = 0.12) relative to saline control mice, along with a decrease in CD11b- DCs (g = -1.06 [95.0%CI -1.94, 0.239], p = 0.0774) and CD8a+ Tcm cells (g = -1.51 [95.0%CI -2.64, -0.263], p 0.0176). In the spleen **(D)**, numerous changes were observed relative to sham saline control animals; Treg treatment increased T helper cells (g = 0.881 [95.0%CI -0.269, 1.85], p = 0.113), FoxP3+ Tregs (g = 1.07 [95.0%CI -0.085, 1.97], p = 0.043), CD11b- DCs (g = 1.09 [95.0%CI -0.257, 2.51], p = 0.0696), and monocytes/macrophages in sham-injured mice (g = 0.853 [95.0%CI -0.293, 1.85], p = 0.134). Similarly, when compared to sham + saline mice, CCI increased T helper cells (g = 0.988 [95.0%CI -0.23, 1.93], p = 0.0756), cytotoxic T cells (g = 1.3 [95.0%CI 0.25, 2.25], p = 0.019), FoxP3+ Tregs (g = 2.34 [95.0%CI 1.37, 3.26], p < 0.0001), and CD8a+ Tcm cells (g = 0.976 [95.0%CI -0.192, 1.93], p = 0.0968).

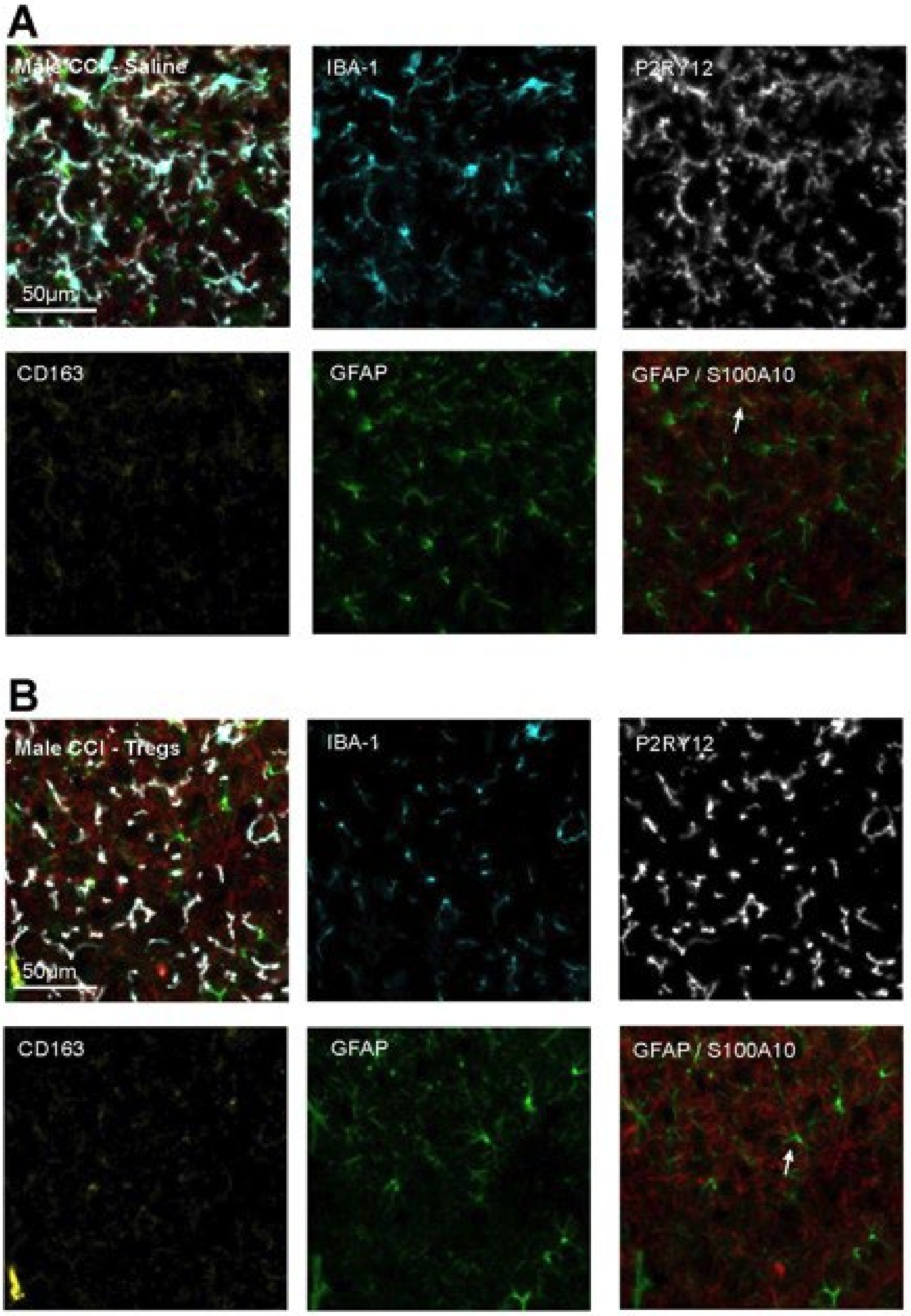

Figure S4. Representative images from Opal multiplex immunohistochemistry highlighting effects of Treg injection in male mice following CCI. Representative images of glial markers taken from the ipsilateral dorsal horn. Top left panel contains immunofluorescence staining of IBA-1 (blue), P2RY12 (white), CD163 (yellow), GFAP (green) and S100A10 (red) in the (A) saline vehicle and (B) Treg-injected male mice (Day 10 post-injury). Arrows indicate GFAP and S100A10 colocalisation.

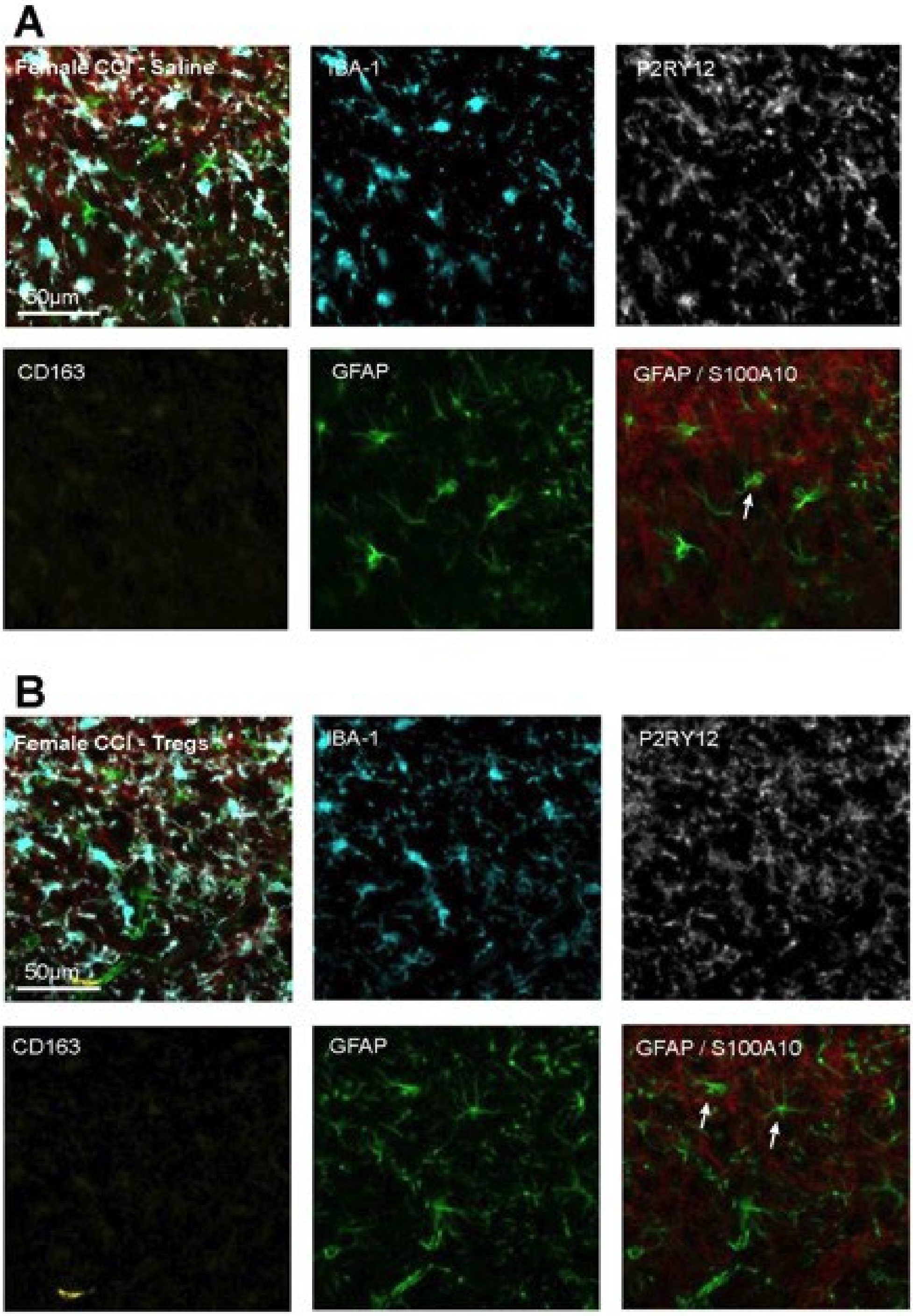

Figure S5. Representative images from Opal multiplex immunohistochemistry highlighting effects of Treg injection in female mice following CCI. Representative images of glial markers taken from the ipsilateral dorsal horn. Top left panel contains immunofluorescence staining of IBA-1 (blue), P2RY12 (white), CD163 (yellow), GFAP (green) and S100A10 (red) in the (A) saline vehicle and (B) Treg-injected female mice (Day 10 post-injury). Arrows indicate GFAP and S100A10 colocalisation.

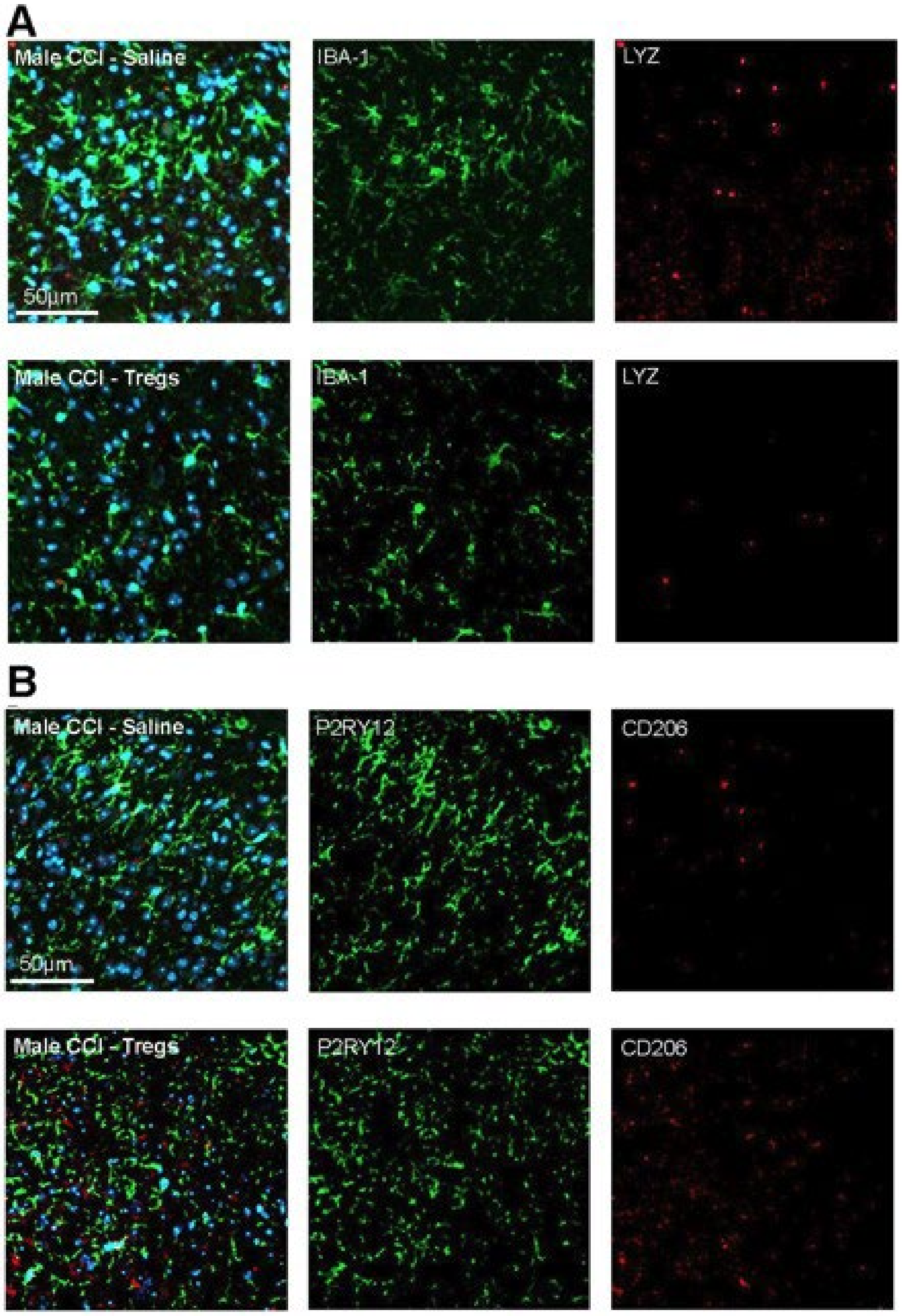

Figure S6. Representative images from immunofluorescent staining highlighting microglial changes following Treg injection in male mice following CCI. Representative images of microglial inflammatory (A) and anti-inflammatory (B) markers taken from the ipsilateral dorsal horn. Left panel contains immunofluorescence staining of (A) IBA-1 (green) and LYZ (red), or (B) P2RY12 (green) and CD206 (red) counterstained with DAPI (blue) in the saline vehicle and Treg-injected male mice (Day 10 post-injury).

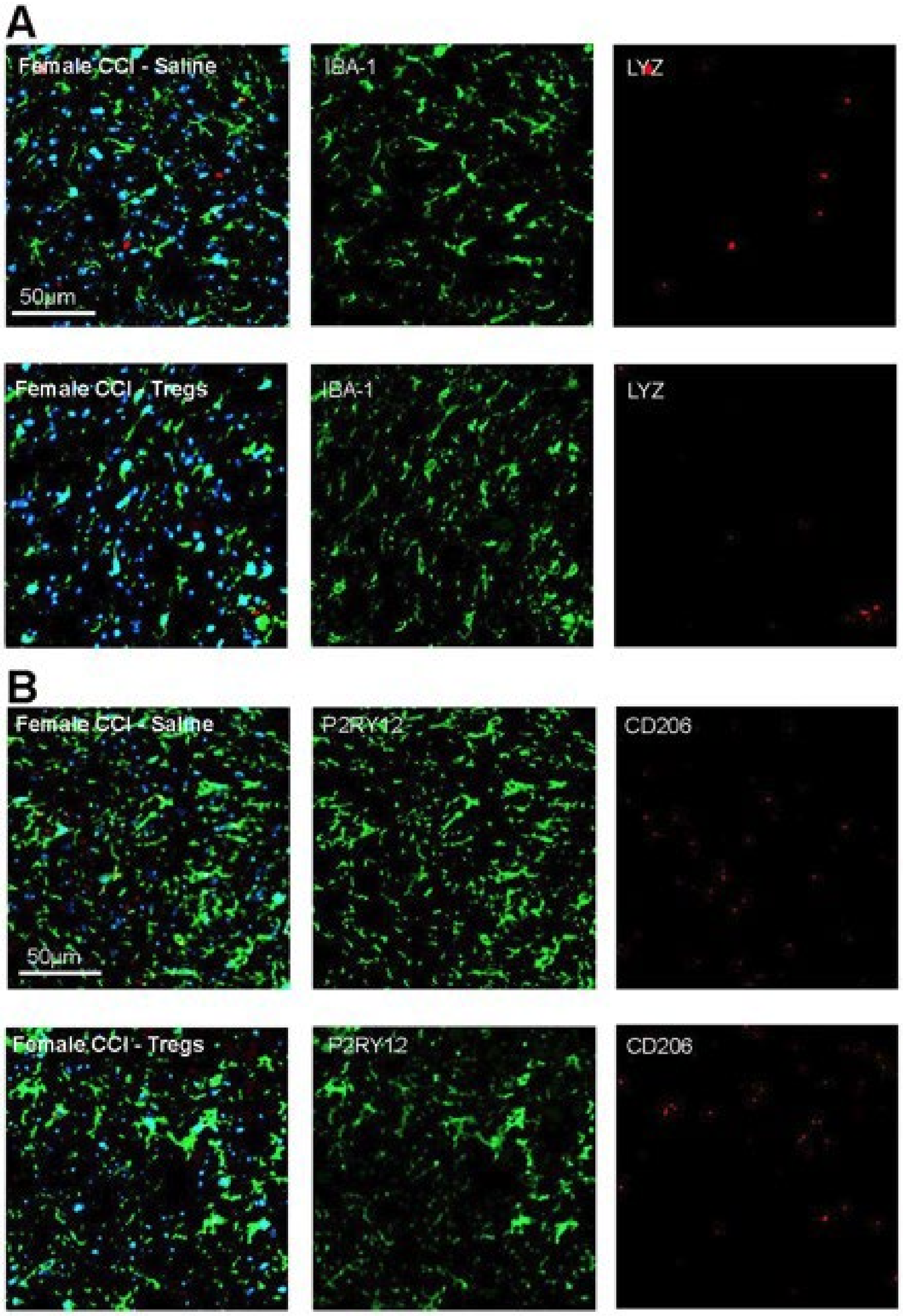

Figure S7. Representative images from immunofluorescent staining highlighting microglial changes following Treg injection in female mice following CCI. Representative images of microglial inflammatory (A) and anti-inflammatory (B) markers taken from the ipsilateral dorsal horn. Left panel contains immunofluorescence staining of (A) IBA-1 (green) and LYZ (red), and (B) P2RY12 (green) and CD206 (red) counterstained with DAPI (blue) in the saline vehicle and Treg-injected female mice (Day 10 post-injury).

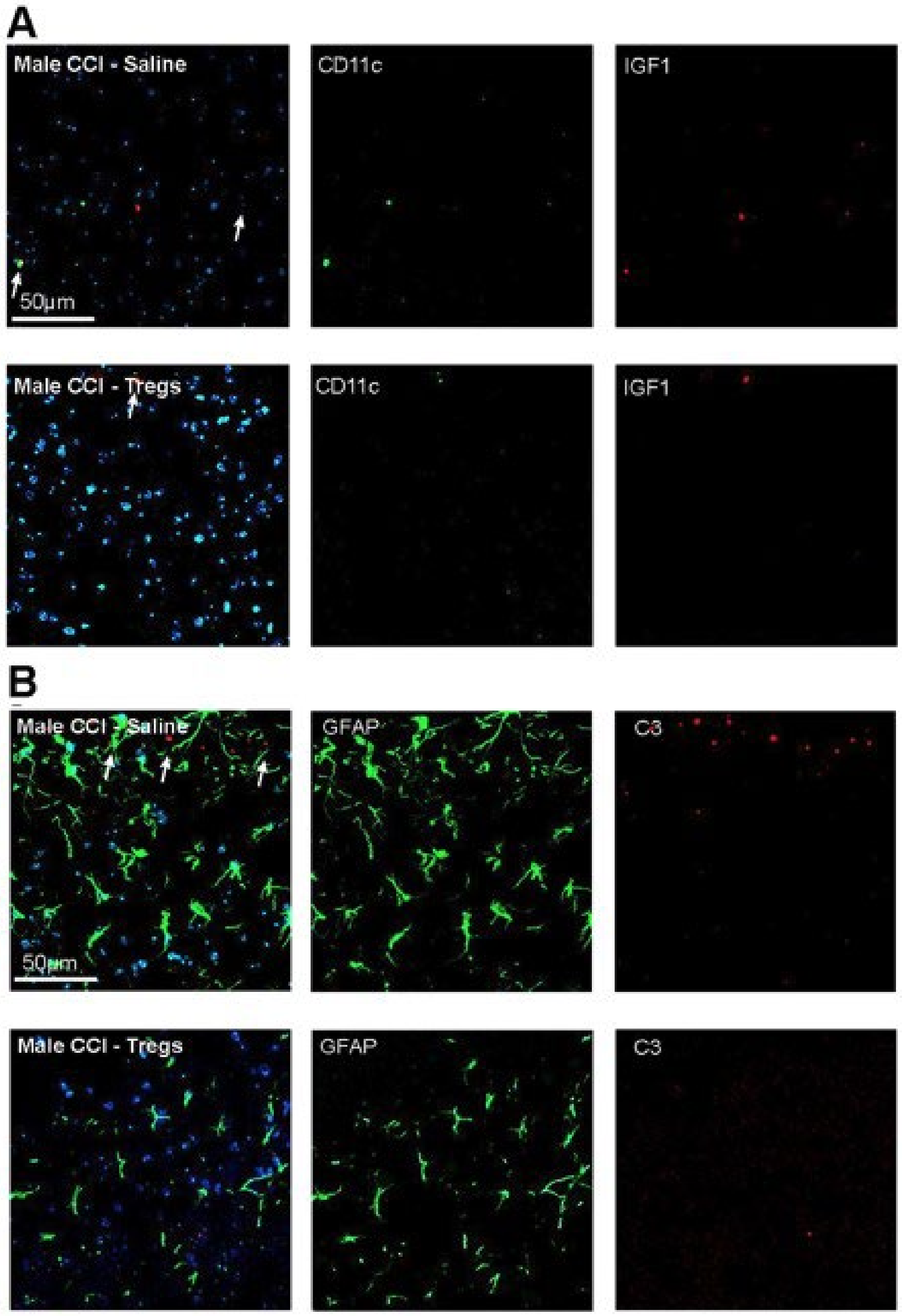

Figure S8. Representative images from immunofluorescent staining highlighting changes in pain-resolving microglia and neurotoxic astrocytes following Treg injection in male mice following CCI. Representative images of pain-resolving microglia (A) and neurotoxic astrocytes (B) markers taken from the ipsilateral dorsal horn. Left panel contains immunofluorescence staining of (A) CD11c (green) and IGF1 (red), and (B) GFAP (green) and C3 (red) counterstained with DAPI (blue) in the saline vehicle and Treg-injected male mice (Day 10 post-injury). Arrows indicate CD11c and IGF1 (A) colocalisation or GFAP and C3 (B) colocalisation.

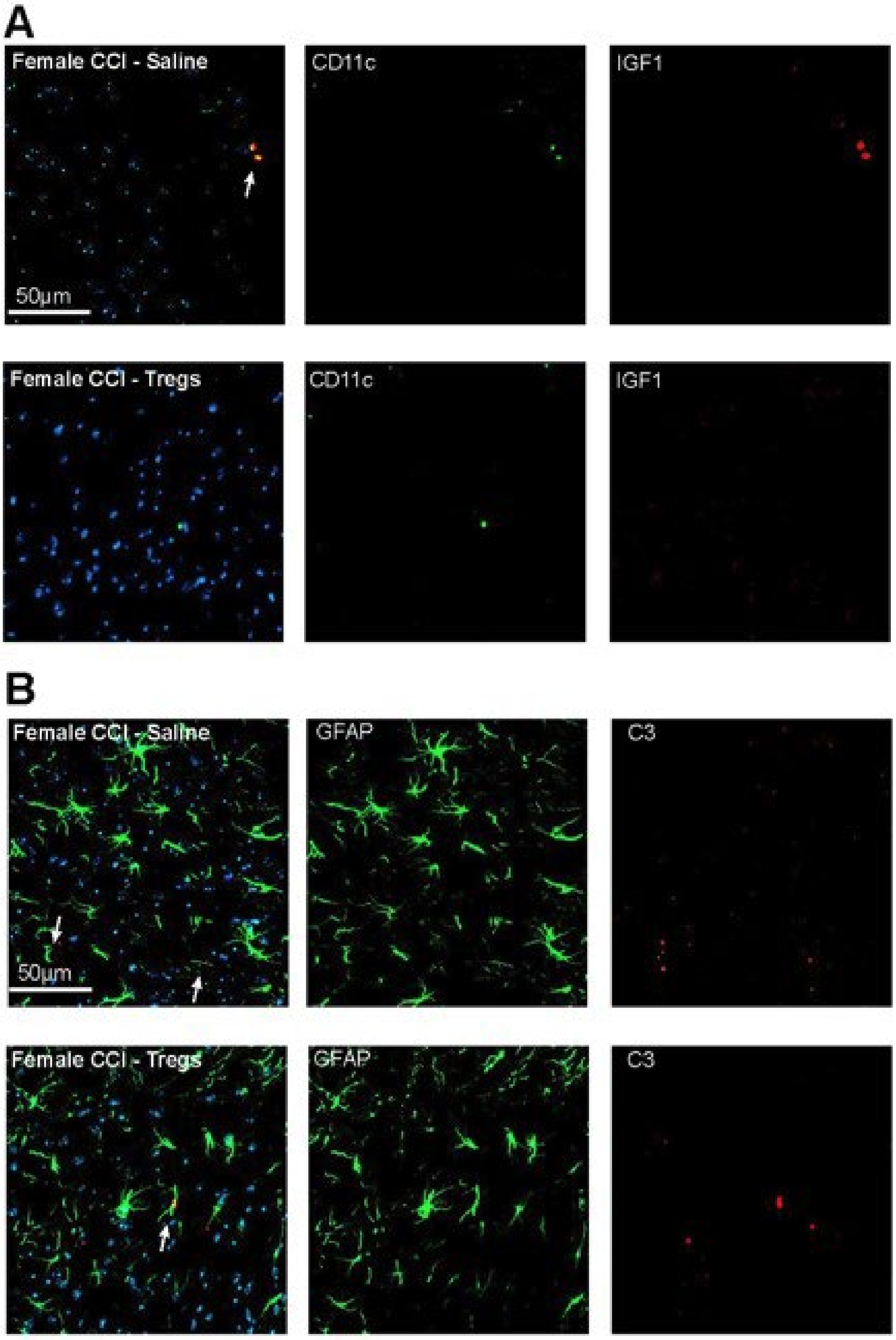

Figure S9. Representative images from immunofluorescent staining highlighting changes in pain-resolving microglia and neurotoxic astrocytes following Treg injection in female mice following CCI. Representative images of pain-resolving microglia (A) and neurotoxic astrocytes (B) markers taken from the ipsilateral dorsal horn. Left panel contains immunofluorescence staining of (A) CD11c (green) and IGF1 (red), and (B) GFAP (green) and C3 (red) counterstained with DAPI (blue) in the saline vehicle and Treg-injected female mice (Day 10 post-injury). Arrows indicate CD11c and IGF1 (A) colocalisation or GFAP and C3 (B) colocalisation.
